# Dysregulated CREB3 cleavage at the nuclear membrane induces karyoptosis-mediated cell death

**DOI:** 10.1038/s12276-024-01195-1

**Published:** 2024-03-13

**Authors:** Ga-Eun Lee, Geul Bang, Jiin Byun, Cheol-Jung Lee, Weidong Chen, Dohyun Jeung, Hyun-Jung An, Han Chang Kang, Joo Young Lee, Hye Suk Lee, Young-Soo Hong, Dae Joon Kim, Megan Keniry, Jin Young Kim, Jin-Sung Choi, Manolis Fanto, Sung-Jun Cho, Kwang-Dong Kim, Yong-Yeon Cho

**Affiliations:** 1 College of Pharmacy, The Catholic University of Korea, Bucheon-si, Gyeonggi-do 14662 Republic of Korea; 2 BK21-4th, and RCD Control∙Material Research Institute, College of Pharmacy, The Catholic University of Korea, Bucheon-si, Gyeonggi-do 14662 Republic of Korea; 3 Research Center for Bioconvergence Analysis, Korea Basic Science Institute, Ochang, Cheongju-si, Chungbuk 28119 Republic of Korea; 4 Research Center for Materials Analysis, Korea Basic Science Institute, Daejeon, 34133 Republic of Korea; 5 Anticancer Agent Research Center, Korea Research Institute of Bioscience and Biotechnology, Cheongju-si, Chungbuk 28116 Republic of Korea; 6 Department of Immunology and Microbiology, School of Medicine, University of Texas Rio Grande Valley, Edinburg, TX 78504 USA; 7 Department of Biology, University of Texas Rio Grande Valley, Edinburg, TX 78539 USA; 8 Department of Basic and Clinical Neuroscience, King’s College London, Maurice Wohl Clinical Neuroscience Institute, London, UK; 9 University of Minnesota, Department of Medicine, 420 Delaware St SE, MMC 284, Minneapolis, MN 55455 USA; 10 Division of Applied Life Science (BK21 four), PMBBRC, Gyeongsang National University, Jinju, 52828 Korea

**Keywords:** Nuclear envelope, Nucleoskeleton

## Abstract

Cancer cells often exhibit resistance to apoptotic cell death, but they may be vulnerable to other types of cell death. Elucidating additional mechanisms that govern cancer cell death is crucial for developing new therapies. Our research identified cyclic AMP-responsive element-binding protein 3 (CREB3) as a crucial regulator and initiator of a unique cell death mechanism known as karyoptosis. This process is characterized by nuclear shrinkage, deformation, and the loss of nuclear components following nuclear membrane rupture. We found that the N-terminal domain (aa 1-230) of full-length CREB3 (CREB3-FL), which is anchored to the nuclear inner membrane (INM), interacts with lamins and chromatin DNA. This interaction maintains a balance between the outward force exerted by tightly packed DNA and the inward constraining force, thereby preserving INM integrity. Under endoplasmic reticulum (ER) stress, aberrant cleavage of CREB3-FL at the INM leads to abnormal accumulation of the cleaved form of CREB3 (CREB3-CF). This accumulation disrupts the attachment of CREB3-FL to the INM, resulting in sudden rupture of the nuclear membrane and the onset of karyoptosis. Proteomic studies revealed that CREB3-CF overexpression induces a DNA damage response akin to that caused by UVB irradiation, which is associated with cellular senescence in cancer cells. These findings demonstrated that the dysregulation of CREB3-FL cleavage is a key factor in karyoptotic cell death. Consequently, these findings suggest new therapeutic strategies in cancer treatment that exploit the process of karyoptosis.

## Redefining cancer treatment: CREB3-driven karyoptosis unveiled

This research investigates the function of a protein, CREB3, in regulated cell death (RCD—a process vital for cell health and disease prevention). The scientists discovered that CREB3 is found in the cell’s nuclear membrane (a barrier protecting the cell’s nucleus) and interacts with DNA. When this protein is split, it can cause the nuclear membrane to break, leading to a type of cell death known as karyoptosis. This process is different from other cell death types like apoptosis and autophagy. The research also found that certain stress factors, such as ultraviolet B radiation and endoplasmic reticulum stress (stress in a cell component that makes proteins), can initiate this process. The results imply that focusing on CREB3 could be a potential cancer treatment strategy. More research is required to further understand these results. This summary was initially drafted using artificial intelligence, then revised and fact-checked by the author.

## Introduction

Regulated cell death (RCD) is cell death that occurs via regulatory cellular systems comprising a variety of biomolecules and is distinguishable from accidental cell death (ACD)^1–3^. Autophagy and apoptosis are classically considered crucial subroutines of RCD that can induce organelle degradation or cell death under cellular stress and that play vital roles in targeted therapy and the regulation of cancer cell death^4–6^, and necroptosis mediated by receptor interacting serine/threonine kinase protein (RIPK) 3/MLKL was also recently classified as a type of regulatory cell death in cancer^7,8^. Since the incidence of malignant tumors increases greatly when excessive cell proliferation occurs or when normal cell death is inhibited, the identification of therapeutic agents that can induce RCD has recently been examined as a new foundation for developing low-toxicity cancer treatment^8,9^. The recent expansion of RCD studies has led to the discovery of many different types of RCD with distinctive characteristics^8^. While accumulating evidence has demonstrated that some genotoxic anticancer drugs, which generally cause nuclear damage and DNA damage resulting in the loss of nuclear integrity can effectively induce RCD in cancer cells^6,10,11^, they cannot be directly used in clinical cancer treatment because of their toxic side effects on normal cells. However, the intrinsic factors and molecular mechanisms that regulate nuclear integrity have not yet been elucidated.

Karyoptosis, a novel type of RCD that exhibits phenotypes such as nuclear shrinkage and atrophy of the cytoplasm^12^, is a recently proposed terminal degradation event associated with autophagy that involves the maladaptive, excessive excretion of nuclear components in neuronal cells^12,13^. Apoptosis and necroptosis are characterized by cell shrinkage, DNA fragmentation, nuclear condensation, necroptosis, cell swelling, organelle swelling, and rupture of the plasma membrane, and karyoptosis can be differentiated from both^12,13^ by the formation of abnormal nuclear morphologies, including herniations, folds and crevices, fragments, and lobules^12–14^. However, the intrinsic factors, extrinsic stimuli, and molecular mechanisms that trigger karyoptosis have not yet been identified.

Cyclic AMP response element-binding protein 3 (CREB3) is an endoplasmic reticulum (ER)/Golgi-bound transcription factor^15,16^. As an activation mechanism in a canonical pathway, cleavage of the transmembrane (TM) domain of wild-type, full-length CREB3 (herein referred to as CREB3-FL) at the Golgi complex (Gc) by S1P and S2P produces the cleaved form of CREB3 (CREB3-CF) and induces its nuclear accumulation^15,17^. Several recent independent studies have focused on CREB3 and sLZIP (herein referred to as CREB3-dTM), an alternative splicing product of CREB3 that does not contain a transmembrane domain and is involved in the responses to ER stress^15^ and Golgi stress^18^ and in cancer cell metastasis^19^. Moreover, as observed by immunocytofluorescence (ICF), abnormal nuclear morphology and nuclear membrane localization frequently accompany ectopic expression of CREB3-FL or CREB3-CF^20,21^. The roles of full-length CREB3 (CREB3-FL) and its cleaved form (CREB3-CF) in the nucleus have not been extensively studied. Here, we present a detailed molecular and mechanistic analysis of a previously unidentified mechanism of cell death termed karyoptosis. We found that karyoptosis is initiated through dysregulation of the transcription factor CREB3. This dysregulation leads to the production and accumulation of CREB3-CF, which promotes karyoptotic cell death in cancer cells and suppresses their proliferation through cellular senescence. Furthermore, our proteomic analysis and screening of extrinsic stimuli suggested that CREB3-CF-mediated karyoptosis is driven by cell death signaling pathways. These pathways are activated by endoplasmic reticulum (ER) stress-mediated nuclear membrane and DNA damage, culminating in cell cycle arrest, cellular senescence, and eventual cell death.

## Materials and methods

### Reagents

Reagents for molecular and cellular biological studies, including MG132 (cat #: C2211) and chloroquine (CQ, cat #: S6999; Selleckchem, Houston, TX, USA), were purchased from Sigma‒Aldrich (Sigma‒Aldrich Korea, Gangnam, Seoul, Korea). Antibodies used for Western blotting, immunoprecipitation (IP), and/or immunocytofluorescence (ICF), including anti-Myc (cat #: sc-40), anti-cullin1 (cat #: sc-17775), anti-γH2AX (cat #: sc-517348), anti-caspase-7 (cat #: sc-81654), anti-Lamin B1 (cat #: sc-6216) and anti-β-actin (cat #: sc-47778), were obtained from Santa Cruz Biotechnology (Dallas, TX, USA). The antibodies anti-caspase-3 (cat #: 9662 S), anti-p62 (cat #: 39749), anti-PARP (cat #: 9532), anti-RIP3 (cat #: 13526 S), anti-RIP3-S227 (cat #: 93654), anti-p53 (cat #: 2527), anti-gasdermin D (cat #: 39754), and anti-caspase-9 (cat #: 9502), were obtained from Cell Signaling Technology (Koram Biotech Corp., Gangnam, Seoul, Korea). Anti-LC3B (cat #: NB100-2220), anti-MLKL-S358 (cat #: ab187091), and anti-caspase-8 (cat #: NB100-56116) antibodies were obtained from Novus Biological (Minneapolis, MN, USA) and Abcam (Woburn, Cambridge, UK), respectively. Anti-CREB3 antibodies were obtained from CUSABIO (cat #: CSB-PA005948, Houston, TX, USA), FineTest (cat #: FNab01962, Wuhan, Hubei, China) or Proteintech (cat #:11275-1-AP, Rosemont, IL, USA). For pulldown experiments, protein G Sepharose beads (cat #: 17-0618-02) were purchased from GE Healthcare (Chicago, IL, USA). Oxaliplatin (Ox, 99% pure) purchased from MedKoo Biosciences (Morrisville, NC 27560, USA) was directly dissolved in 10× PBS (25 μM stock solution). Fresh stock solution was prepared before addition to the cell culture medium and utilized within 24 h.

### Cell culture

HEK293T and HeLa cells were cultured in Dulbecco’s modified Eagle’s medium (DMEM, cat #: 10-013-CV; Corning Korea, Seoul, Korea), and SK-MEL-2 cells were cultured in minimum essential medium supplemented with Eagle medium, Earle’s salts & L-glutamine (MEM, cat #: 10-010-CV; Corning Korea) and 10% fetal bovine serum (FBS, cat#: 35-015-CV; Corning Korea). All cells were maintained at 37 °C in a 5% CO_2_ incubator and passaged at approximately 90% confluence.

### Expression vectors

Myc- and Flag-tag fusion proteins were constructed by basic recombinant DNA technology using pCMV-Myc from TAKARA Bio INC. (cat #: 635689, Kusatsu, Shiga, Japan) and pBICEP-CMV-2 Flag from Sigma‒Aldrich (cat #: E0904, Sigma‒Aldrich Korea, Gangnam, Seoul), respectively. mCherry-Lamin B1 (cat #:55069) and mCherry-Lamin A (cat #:55068) expression vectors were purchased from Addgene (Watertown, MA, USA). To establish cells stably expressing CREB3-CF, CREB3-dTM (also known as sLZIP), or CREB3-CF-dbZIP in SK-MEL-2 cells and HeLa cells, the pCDH-CMV-MCS-EF1-puro viral vector was purchased from Addgene. The expression vectors utilized in this study were confirmed by DNA sequencing before use.

### Western blot and immunoprecipitation

Cell lysates (20 μg) were extracted with RIPA buffer containing 1% Triton X-100, 0.1% SDS, 0.5% sodium deoxycholate, 50 mM Tris-HCl (pH 7.4), 150 mM NaCl, and 2 mM EDTA, resolved by SDS‒PAGE, transferred onto PVDF membranes, and hybridized with specific antibodies as indicated. For immunoprecipitation, total lysates (150 μg) were incubated with specific antibodies (2 mg/ml) as indicated at 4 °C for 4 h or overnight. The target proteins were immunoprecipitated by the addition of protein G Sepharose beads (GE Healthcare), incubation at 4 °C for 2 h, and centrifugation. The proteins bound to the beads were washed using washing buffer (20 mM Tris at pH 8.0, 100 mM NaCl, 1 mM EDTA, and 0.5% NP-40), boiled, and visualized by Western blotting as described above.

### Ectopic expression and gene knockdown

HEK293T cells were used to produce viral particles by using lentiviral or retroviral packaging systems. In brief, overexpression or knockdown viral vectors were cotransfected with packaging system vectors into HEK293T cells following the guidelines provided by Addgene, after which the cells were cultured for 24–48 h at 37 °C in a 5% CO_2_ incubator. The medium was collected, filtered using 0.45 μm acetate syringe filters, and used to infect SK-MEL-2 or HeLa cells. Infection was conducted by spreading a combined mixture containing the appropriate amount of viral particles and polybrene (final concentration of 1 μg/ml; Sigma‒Aldrich) for 4–6 h, after which the mixture was cultured overnight to stabilize the cells. The cells were then treated with puromycin (5 μg/ml) for 2–3 days, pooled, and utilized for the following experiments. The efficacy of overexpression or gene knockdown was confirmed by Western blotting.

### DNA association assay

To confirm the DNA association of CREB3, we selected RIPA buffer containing 1% Triton X-100, 0.1% SDS, 0.5% sodium deoxycholate, 50 mM Tris-HCl (pH 7.4), 150 mM NaCl, and 2 mM EDTA. Briefly, SK-MEL-2 or HeLa cells stably or transiently expressing mock, CREB3-FL, CREB3-CF, or CREB3-sLZIP were harvested, washed, and suspended in RIPA buffer. The suspension was sonicated for 15 cycles of 30 s at full power and a 30 s resting interval to solubilize proteins, including integral membrane proteins. The solubilized and chromatin-bound proteins were separated by centrifugation at 13,000 rpm for 5 min at 4 °C, and appropriate concentrations of proteins were utilized to detect chromatin-unbound and chromatin-bound CREB3 proteins by Western blotting.

### Confocal microscopy

The cells that stably or transiently expressed Myc-CREB3-CF, the mutants, mCherry-EGFP-LC3B, mCherry-Lamin A or mCherry-Lamin B1 were stained with subcellular dyes specific for the Golgi complex, ER, mitochondria, and nuclei and subjected to confocal microscopy. The cells cultured in chamber slides were fixed, permeabilized using 0.5% Triton X-100/1× PBS, and blocked with 1× PBS/0.02% Tween 20/1% BSA at 37 °C for 1 h. To detect protein expression, the cells were hybridized with anti-Myc (cat #: sc-40; Santa Cruz; cat#: 2272; Cell Signaling Technology) or γH2AX (cat #: sc-517348; Santa Cruz) overnight at 4 °C as indicated and then hybridized with Alexa Fluor 488-conjugated goat anti-mouse (cat#: A-11029; Invitrogen, Waltham, MA, USA), Alexa Fluor 488-conjugated goat anti-rabbit (cat#: A-11029; Invitrogen), Alexa Fluor 568-conjugated goat anti-mouse (cat#: A-11031, Invitrogen); or Alexa Fluor 647-conjugated goat anti-mouse (cat#: A-21235; Invitrogen) at RT for 1 h. Fluorescence was observed under an LSM 710 laser scanning confocal microscope (Carl Zeiss Korea Co. Ltd., Seoul, Korea), and fluorescence intensity was measured using ImageJ ver. 1.53a (National Institutes of Health, Bethesda, MD, USA).

### Transmission electron microscopy

Subcellular ultrastructures were observed via transmission electron microscopy. Briefly, cells were fixed in 2.5% glutaraldehyde for 2 h at 4 °C, washed with 0.1 M phosphate buffer, and fixed in 1% (wt/vol) OsO_4_ in phosphate buffer. The cells were dehydrated through 50∼100% ethanol for 15 min and embedded in Embed 812 Epoxy resin. After polymerization of the resin at 70 °C for 48 h, serial sections were cut and mounted on formvar-coated slot grids. Sections were stained with 2% (wt/vol) uranyl acetate for 20 min and with lead citrate for 5 min. The subcellular ultrastructure of the cells was observed under a transmission electron microscope (Tecnai G2 Spirit Twin; FEI Company; Korea Basic Science Institute).

### In vitro pulldown assay

His-CREB3-CF fusion proteins were partially purified by 0.5 mM IPTG induction for 4 h at 37 °C, subjected to ultrasonication lysis, and pulled down using HisPur Ni-NTA resin (cat #: 88221; Thermo Scientific^TM^, Waltham, MA, USA). The purified His-CREB3-CF proteins were combined with HEK293T cell lysates containing transiently expressed mCherry-Lamin A or mCherry-Lamin B1 and incubated overnight at 4 °C. The HisPur Ni-NTA resin was washed three times with ice-cold wash buffer (100 mM NaCl, 20 mM Tris (pH 8.0), 1 mM EDTA, and 0.5% NP-40) and precipitated by centrifugation (1000 × *g*) at 4 °C. The precipitates were resolved by SDS‒PAGE, and the indicated proteins were visualized by Western blotting.

### TMT 18-plex labeling and LC‒MS/MS analysis

Trypsin-digested peptides were labeled using 18-plex TMT reagent according to the manufacturer’s instructions (Thermo Fisher Scientific, Inc., MA, USA). Peptides from the 5 different samples obtained from mock, CREB3-FL, and CREB3-CF stable cells were labeled with TMT reagents. All the labeled peptides were combined, and high-pH reversed-phase liquid chromatography (RPLC) fractionation was subsequently carried out using a NexeraXR HPLC system (Shimadzu Corp., Kyoto, Japan). Briefly, the desalted peptide mixture was injected onto an Xbridge C18 column (4.6 × 250 mm, 5 µm) and fractionated into 20 fractions using high-pH buffer A (10 mM ammonium formate in water, pH 10.0) and buffer B (10 mM ammonium formate in 90% acetonitrile, pH 10.0). All the fractions were collected using an FRC-10 (Shimadzu) fraction collector. The separated peptides were collected noncontiguously, dried in a speed vacuum, and stored at −80 °C. Peptides were analyzed using an LC‒MS/MS system consisting of an UltiMate 3000 RSLCnano system (Thermo Fisher Scientific) and an Orbitrap Eclipse Tribrid mass spectrometer (Thermo Fisher Scientific) equipped with a nano-electrospray source (EASY-Spray Sources, Thermo Fisher Scientific). Peptides were trapped in a 75 μm × 2 cm C18 precolumn (nanoViper, Acclaim PepMap100, Thermo Fisher Scientific) before being separated on an analytical C18 column (75 μm × 50 cm PepMap RSLC, Thermo Fisher Scientific) with a flow rate of 250 nL/min and a total run time of 95 min. Mobile phases A and B consisted of 100% water containing 0.1% formic acid and 100% acetonitrile (ACN) containing 0.1% formic acid, respectively. During chromatographic separation, the Orbitrap mass spectrometer was operated in data-dependent mode, automatically switching between MS1 and MS2. The MS data were acquired using the following parameters: Full-scan MS1 spectra (400–1600 m/z) were acquired in the Orbitrap for a maximum ion injection time of 50 ms at a resolution of 120,000 and a standard mode automatic gain control (AGC) target. MS2 spectra were acquired with an Orbitrap mass analyzer at a resolution of 30,000 with the turbo-TMT setting by applying high-energy collision dissociation (HCD) with a 36% normalized collision energy and an AGC target value of 5.0 × 10^4^ with a maximum ion injection time of 54 ms. Previously fragmented ions were excluded for 20 s. Identification and quantification of MS/MS spectra were performed using Integrated Proteomics Pipeline software with the UniProt human database (released on Apr-07-2023).

### Holotomography

To measure the dynamic membrane fluctuations associated with karyotosis, a commercial holotomography system (HT-X1; Tomocube, Inc., Daejeon, Republic of Korea) was used as previously described^22^. A diode-pumped solid-state laser (wavelength, λ = 532 nm) was used as the illumination source. The beams from the laser source were split into two arms: one for the sample and the other for the reference. The sample beam is reflected from a digital micromirror device (DMD), and the angle of the beam impinging onto a sample is systematically controlled by projecting hologram patterns on the DMD. The diffracted beam from a sample is projected onto an image plane, where the sample beam interferes with the reference beam and generates holographic patterns. The phase and amplitude information were retrieved using a phase retrieval algorithm from multiple 2-D holograms measured at various illumination angles (*N* = 49). Then, the 3D RI tomogram of a cell, *n*_karyoptosis_(*x*, *y*, *z*), was reconstructed using an algorithm of optical diffraction tomography. The time-lapse 2D holograms were measured at a frame rate of 100 Hz. From the time-lapse 2D holograms, the optical phase delay Δ*ϕ*(x, *y*, *t*) of a cell undergoing karyoptosis were measured for 256 timepoints with a frame rate of 150/s. Then, the dynamic cell height was calculated as Δ*h*(*x*, *y*, *t*)=Δ*ϕ*(*x*, *y*, *t*)· *λ*/(〈*n*_karyoptosis_〉−*n*_m_)/2π, where 〈*n*_karyoptosis_〉 is the mean RI of a cell undergoing karyoptosis, and nm is the RI of a given medium. Then, the dynamic karyoptotic cell-MFs were calculated as the root-mean-square of the height difference, Σ[Δ*h*(*x*, *y*, *t*)−〈Δ*h*(*x*, *y*, *t*)〉] 2/*N*_frame_, where *N*_frame_ is the total number of frames. The detailed procedures can also be found elsewhere.

### Statistical information

The data are expressed as the mean ± standard error of the mean (SEM). GraphPad Prism 8 was used for statistical analysis. The sample sizes, reproducibility of the experiments and statistical tests used are presented in the figure legends. ZEN 3.3 on a Zeiss LSM 710 confocal laser microscopy system was used for immunohistochemistry data collection and analysis. A Bio-Rad ChemiDoc Touch was used for Western blot data collection and analysis. BD Biosciences BD CellQuest Pro software on a FACSCalibur or ModiFit LT (ver3.3) was used for flow cytometry data collection and analysis.

## Results

### CREB3 is a nuclear membrane protein

**Fig. 1:**
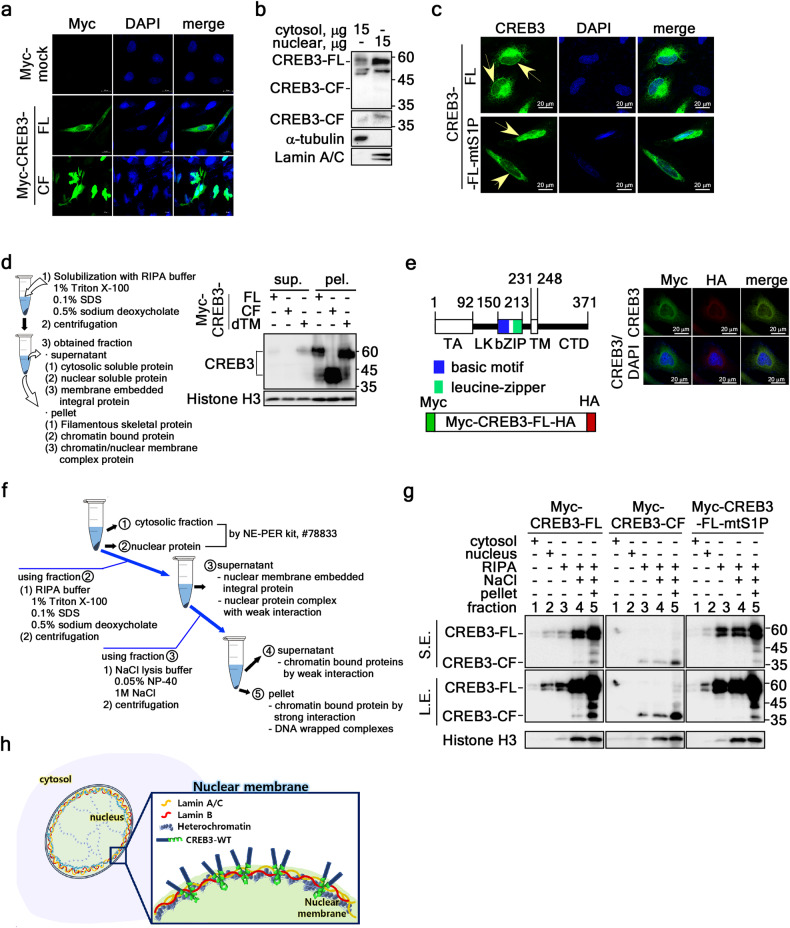
CREB3-FL is directly localized to the nuclear membrane and not to the canonical ER/Gc transport or S1P/S2P cleavage pathway. **a** Illustration showing the localization of CREB3-FL and CREB3-CF and changes in nuclear morphology. **b** Western blotting showing the localization of endogenous CREB3-FL and CREB3-CF in the cytosolic and nuclear fractions. **c** Illustration showing the nuclear membrane localization of CREB3-FL and CREB3-FL-mtS1P. **d** Left panels: Diagram showing the experimental strategies used to obtain the extracts of the cytosolic and nuclear fractions. Right panels, Western blotting showing nuclear DNA bound to CREB3-FL, CREB3-CF, and CREB3-dTM. **e** Confocal microscopy image illustrating the nuclear membrane localization of the Myc-CREB3-FL-HA double-tagged protein. **f** Diagram showing the experimental strategy used to determine the extent of nuclear DNA binding to CREB3-FL, CREB3-CF, and CREB3-FL-mtS1P. **g** Western blotting showing nuclear DNA binding of CREB3-FL, CREB3-CF, and CREB3-FL-mtS1P in the presence of NaCl. **h** Illustration of the localization and orientation of CREB3 at the nuclear membrane.

Canonically, CREB3-FL is a type II membrane protein that is cleaved by S1P/S2P at the Golgi complex (Gc)^23^, producing CREB3-CF^24^. Although CREB3 is known as a transcription factor belonging to the bZIP superfamily^23^ that positively or negatively regulates the expression of target genes, such as ARF4^18,25^ and PPARγ_2_^25^, the effect of CREB3 in cancer cells requires further elucidation. To examine the role of CREB3 in cancer cell proliferation, we introduced CREB3-FL and CREB3-CF (Supplementary Fig. 1a) into SK-MEL-2 melanoma cells. Surprisingly, morphological changes (referred to here as fragility) such as nuclear DNA herniation, nuclear lobulation, and nuclear membrane ripping, were observed (Fig. 1a). Although CREB3-FL overexpression increased DNA herniation and led to abnormal nuclear morphology in some cells, CREB3-FL was observed to induce less fragility (Fig. 1a). In previous work, CREB3L1 (known as Oasis, a member of the CREB3 subfamily) was shown to localize to the nuclear membrane^26^, which suggested that CREB3-FL might play a functional role in the nuclear membrane. Western blotting of the cytosolic and nuclear fractions showed that endogenous CREB3-FL and CREB3-CF were mainly detected in the nuclear fraction (Fig. 1b). The presence of CREB3-FL at the nuclear membrane was also confirmed by confocal microscopy using CREB3-FL and CREB3-FL-mtS1P, which is the S1P cleavage site mutant (Fig. 1c and Supplementary Fig. 1b). Given that CREB3 interacts with DNA through its basic leucine zipper (bZIP) domain^23^, determining whether CREB3-FL is DNA bound was necessary to ascertain whether CREB3-FL is directly localized to the nuclear membrane. To investigate this possibility, we extracted two types of proteins using RIPA buffer supplemented with 1% Triton X-100, 0.1% SDS, and 0.5% SDOC (Fig. 1d). The first type included soluble proteins, encompassing both soluble and membrane-bound proteins in the cytosol and nucleus, along with protein complexes that interact weakly and integral membrane integral proteins. This type was designated as the supernatant. The second type consisted of insoluble pelleted proteins, encompassing filamentous cytoskeletal and nuclear-skeletal proteins, as well as DNA-associated chromatin-bound proteins (designated as the pellet). The CREB3 proteins, which included CREB3-FL, CREB3-CF, and CREB3-dTM (Supplementary Fig. 1c), were detected mainly in the pellet fraction (Fig. 1d), indicating that CREB3-FL is directly localized to the nuclear membrane. This conclusion was supported by the evidence that Myc- and HA-tagged CREB3 at the N- and C-termini, respectively, were similarly localized to the nuclear membrane (Fig. 1e). To determine whether the intact CREB3-FL at the nuclear membrane forms a complex with genomic DNA, we devised an assay of CREB3-FL dissociation from chromatin in the pellet using 1 M NaCl (Fig. 1f). The strategy revealed that CREB3-FL, CREB3-CF, and CREB3-FL-mtS1P bind strongly to genomic DNA (Fig. 1g). Based on these results, we proposed a model in which CREB3-FL resides at the nuclear inner membrane, the N-terminal 1-230 region of CREB3 interacts with nuclear DNA, and the C-terminal half of CREB3-FL might localize to the intermembrane space between the inner and outer nuclear membranes (Fig. 1h). Our data suggested that CREB3-FL localizes to the nuclear membrane, where it interacts with genomic DNA via CREB3-CF.

### Dysregulation of CREB3-FL cleavage ruptures the nuclear membrane

**Fig. 2:**
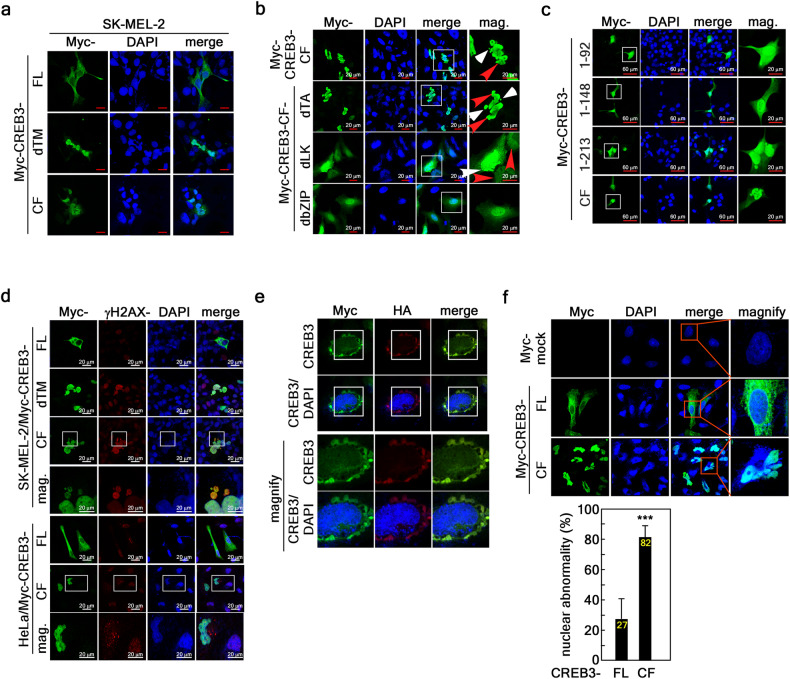
Dysregulation of CREB3-FL cleavage results in tearing of the nuclear membrane. **a** Illustration showing the relationship between the localization of CREB3-FL, CREB3-dTM and CREB3-CF and changes in nuclear morphology. **b** ICF images showing the relationship between domain deletion of CREB3-CF and changes in nuclear morphology. **c** ICF images showing the relationship between the serial deletion of CREB3-CF and changes in nuclear morphology. **d** ICF images showing the effects of CREB3-FL, CREB3-CF, and CREB3-dTM on DDR, DNA herniation, and microsatellite formation. **e** Illustration showing Myc-CREB-FL-HA at the inner nuclear membrane and nuclear membrane ripping and aggregation via karyoptosis. **f** Illustration of the frequency of nuclear abnormalities in relation to CREB3-CF expression. The error bars indicate the SEMs. ***P < 0.0001 (Student’s *t* test).

Since the nuclear accumulation of CREB3-CF induces nuclear fragility (Fig. 1a), the anchoring of CREB3-FL to the nuclear membrane via the TM might be vital for maintaining nuclear integrity. This hypothesis was supported by the finding that CREB3-dTM induced nuclear fragility similar to that of CREB3-CF (Fig. 2a). Moreover, the bZIP domain deletion of CREB3-CF (CREB3-CF-dbZIP) converted the nuclear shape from the fragile form to the round form (Fig. 2b). However, CREB3-CF-dbZIP was detected in the nucleus (Fig. 2b, c and Supplementary Fig. 2a, b). Taken together, these results indicated that DNA binding to CREB3-FL via the bZIP domain is indispensable for nuclear integrity. Since CREB3-CF-induced nuclear fragility also led to dispersion and punctate DAPI staining in the cytosol (Figs. 1a and 2a–c), the loss of nuclear integrity might induce a DNA damage response (DDR). ICF analysis with a γH2AX antibody indicated that CREB3-CF, γH2AX, and DAPI colocalized with microsatellites (Fig. 2d, upper panels) or in the cytoplasm (Fig. 2d, bottom panels), supporting this hypothesis. The robust detection of γH2AX indicated that the nuclear membrane might be ruptured or ripped. Tearing of the nuclear membrane was observed by high magnification confocal microscopy following Myc-CREB3-FL-HA overexpression (Fig. 2e). To compare the effects of CREB3-FL and CREB3-CF on nuclear fragility, we counted the number of cells with nuclear fragility among the Myc-positive cells. The results demonstrated that approximately 82% of the CREB3-CF-overexpressing cells showed the nuclear fragility, while only 28% of the CREB3-FL cells showed nuclear fragility (Fig. 2f). The results demonstrated that the DNA binding and nuclear membrane anchoring of CREB3-FL are indispensable for maintaining nuclear integrity.

### CREB3-CF-induced nuclear membrane ripping induces karyoptosis

**Fig. 3:**
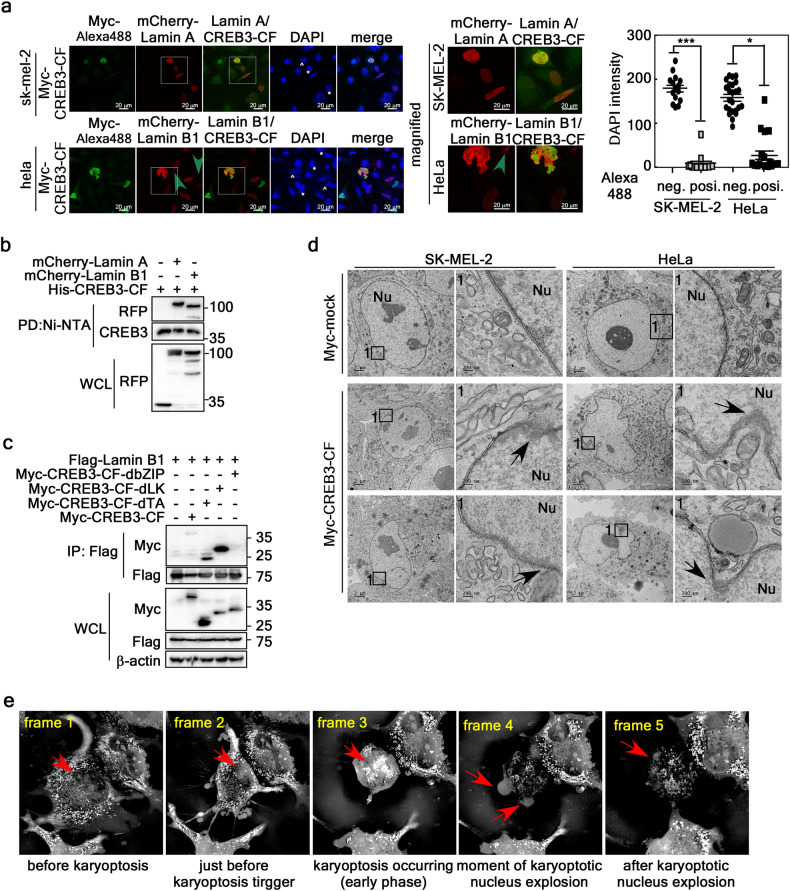
CREB3-CF-induced nuclear membrane ripping initiates karyoptosis. **a** Illustration of nuclear membrane deformation-induced nuclear DNA loss. Left panels, Illustration of CREB3-CF-induced nuclear membrane deformity and loss of nuclear DNA. Central panels. Magnification of the boxed area in the left panels. Graphs, Comparison of DAPI intensity between CREB3-CF-negative and CREB3-CF-positive cells (*n* = 56–87 cells). The error bars indicate the SEMs. *P < 0.05, ***p < 0.0001 (Student’s *t* test). **b** IP/Western blots showing the interaction of Lamin A and B1 with CREB3-CF. **c** Illustration of the interaction between the bZIP domain of CREB3-CF and Lamin B1. **d** Transmission electron microscopy images showing nuclear membrane abnormalities in terms of shape and integrity in mock- and CREB3-CF-overexpressing cells. *Arrows* indicate nuclear membrane invagination and membrane integrity loss. **e** Illustration of explosive nuclear membrane rupture during karyoptosis. Frame 1, image showing normal nuclear morphology before karyoptosis initiation; Frame 2, image showing initiation of karyoptosis; Frame 3, image of early-stage karyoptosis; Frame 4, image of the moment of karyoptotic nucleus explosion (karyoptosis); Frame 5, image after karyoptotic nucleus explosion. The live and time-lapse videos are in Supplementary Live Images, Supplementary Live Image 1–3.

CREB3-CF-induced nuclear membrane ripping resulted in abnormal nuclear morphology and DDR (Fig. 2a, c, d, f). A similar phenomenon is observed in progeria syndrome, a genetic disease caused by abnormal Lamin polymerization^27^. CREB3-CF overexpression increased the Lamin intensity (Fig. 3a, left panels) and led to abnormal folding and cytosolic Lamin puncta (Fig. 3a, right panels). Interestingly, when the DAPI intensities of the CREB3-CF-positive and CREB3-CF-negative cells were compared, the CREB3-CF-overexpressing cells clearly had lower DAPI intensity than the CREB3-CF-negative cells (Fig. 3a, graphs). Since CREB3-FL is anchored to the nuclear membrane and the bZIP domain of CREB3-FL interacts with genomic DNA, the N-terminal 1-230 region of CREB3 may interact with Lamin. This hypothesis was examined by a Ni-NTA pulldown assay using His-CREB3-CF and mCherry-Lamin A or mCherry-Lamin B1, which indicated that Lamin A and Lamin B1 interacted (Fig. 3b). The interaction domain of CREB3-CF was determined by IP using Myc-CREB3-CF serial deletion mutants (Supplementary Fig. 2b), which indicated that the bZIP domain of CREB3 interacted with Lamin B1 (Fig. 3c). To clarify the nuclear morphological alterations induced by CREB3-CF overexpression, we further observed the ultrastructures of subcellular organelles via TEM. SK-MEL-2 cells and HeLa cells stably expressing Myc-mock had a normal nuclear ultrastructure with well-dispersed chromatin, an intact nuclear membrane, and other subcellular organelles, including mitochondria (Fig. 3d, top panels). In contrast, SK-MEL-2 cells and HeLa cells stably expressing CREB3-CF exhibited striking morphological changes in the nucleus, with elongation, twisted/wrinkled nuclei, vesicles in the nucleus, and invagination of the nuclear membrane (Fig. 3d). Moreover, CREB3-CF overexpression in SK-MEL-2 and HeLa cells led to ruptured nuclear membranes (Fig. 3d, arrows in the middle and bottom panels). Interestingly, the invaginated vesicular areas observed in the nuclei of both SK-MEL-2 and HeLa cells overexpressing CREB3-CF contained high-density heterochromatin (Fig. 3d, arrows in the middle and bottom panels). By holotomography using SK-MEL-2 cells stably overexpressing mock, CREB3-FL, or CREB3-CF, we recorded live images for explosive nuclear rupture (Fig. 3e and Supplementary Live Image 1–3). SK-MEL-2 cells stably expressing CREB3-CF initially exhibited a normal nuclear morphology (Fig. 3e, frame 1). However, the cells subsequently underwent nuclear shrinkage (Fig. 3e, frame 2), and nuclear condensation (Fig. 3e, frame 3). In frame 3, the cytoplasm was flattened and attached to the vessel surface. After this, the nucleus explosively ruptured (Fig. 3e, frame 4). Live images at the time of nuclear rupture clearly showed that the nuclear membrane initially formed several small nuclear membrane vesicles, and then an explosive wave of nuclear rupture spread outward, likely as a spray of water. The nuclear materials at the rupture sites collapsed and were engulfed by neighboring cells (Fig. 3e, frame 5). Since these cells underwent explosive nuclear rupture, we referred to this type of cell death as karyoptosis.

### Karyoptosis is distinguishable from apoptosis, autophagy, necroptosis, and pyroptosis

**Fig. 4:**
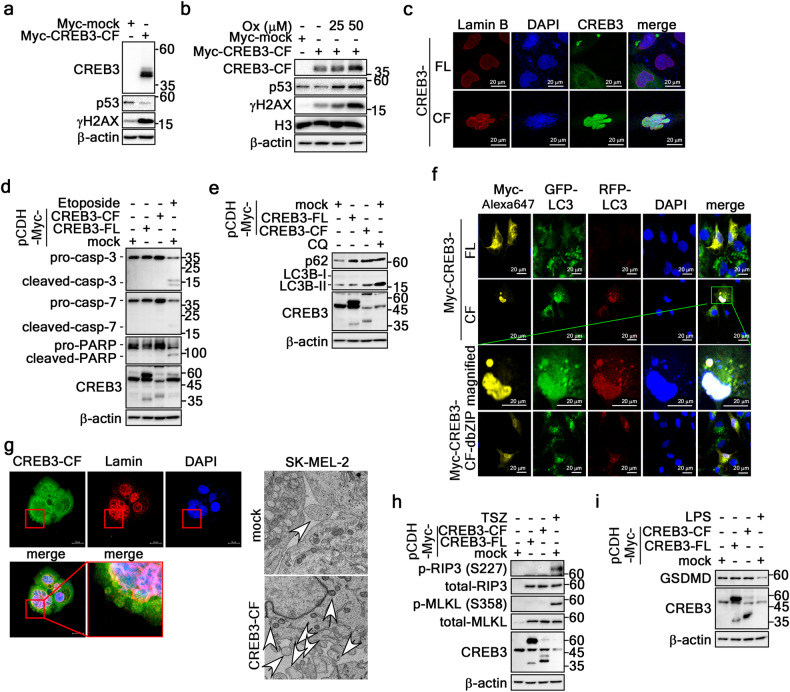
Karyoptosis is distinguishable from apoptosis, autophagy, necroptosis and pyroptosis. **a** Illustration showing that CREB3-CF-induced karyoptosis evokes DDR, indicating an increase in γH2AX. **b** Illustration showing that CREB3-CF-induced karyoptosis is decoupled from the p53-dependent DDR signaling pathway. **c** ICF images showing that loss of CREB3 anchoring at the nuclear membrane induces nuclear lobulation, invagination, and nuclear DNA herniation. **d** Western blots indicating that CREB3-CF-induced karyoptosis does not induce the cleavage of apoptosis markers. **e** Western blots indicating that CREB3-CF-induced karyoptosis inhibits autophagy. **f** ICF analysis showing that CREB3-CF overexpression increased the nuclear accumulation of LC3. **g** ICF image showing that CREB3-Cf increases the formation of extracellular vesicles containing CREB3-CF, lamin, and genomic DNA. Right panels, TEM images illustrating that CREB3-CF overexpression induces abnormal nuclear morphology and increases extracellular vesicle formation. **h** Western blots showing that CREB3-CF-induced karyoptosis differs from necroptosis. **i** Western blots showing that CREB3-CF-induced karyoptosis is distinguishable from pyroptosis.

CREB3-CF expression increased the number of γH2AX puncta in the nucleus and cytoplasm (Fig. 2d), indicating that karyoptosis might be associated with DDR. Unexpectedly, we found that the p53 protein level, which is canonically increased by DDR^28^, was decreased by CREB3-CF expression (Fig. 4a). Notably, oxaliplatin, a DNA damaging anticancer drug, counteracted the CREB3-CF-induced decrease in the p53 protein level (Fig. 4b). Moreover, the CREB3-CF-induced increase of the γH2AX protein was more increased by oxaliplatin treatment (Fig. 4b). Karyoptotic nuclear rupture was observed by ICF using an anti-Lamin B antibody and DAPI staining, indicating that CREB3-CF induced nuclear fragility and herniation of nuclear DNA (Fig. 4c). These results suggested that the CREB3-CF-induced DDR differs from the extrinsic DDR induced by anticancer drugs, which is a hallmark of DNA damage-mediated apoptosis^29^. Based on our hypothesis, we observed that overexpressing CREB3-FL or CREB3-CF did not affect the cleavage of caspase-7, caspase-3, or poly(ADP-ribose) polymerase (PARP). In contrast, treatment with etoposide affected the expression of these factors (Fig. 4d), indicating that CREB3-CF-induced karyoptosis is distinct from apoptosis. Additionally, overexpressing either CREB3-FL or CREB3-CF increased the levels of p62 and cleaved LC3B-II, similar to the effects observed with chloroquine treatment, indicating that overexpression of CREB3-FL and CREB3-CF inhibits autophagy (Fig. 4e). These findings were further supported by the ICF data showing that CREB3-CF overexpression led to increased nuclear accumulation of LC3, which is not typically observed in canonical autophagy. Notably, CREB3-CF induced the formation of microsatellites, which were contained with CREB3-CF, LC3, and DAPI (Fig. 4f, magnified panels). In contrast, the overexpression of CREB3-FL or CREB3-CF-dbZIP showed complete dissociation of the DAPI, LC3, and CREB3 staining results (Fig. 4f, *top* and bottom panels). The microsatellites that formed due to CREB3-CF overexpression (Fig. 4g, ICF in the left panels) were associated with an increase in extracellular vesicles (Fig. 4g, TEM in the right panels), strongly suggesting the mechanism of that CREB3-CF-induced karyoptosis is distinct from canonical autophagy. Furthermore, neither CREB3-FL nor CREB3-CF overexpression increased the phosphorylation of RIP3 or MLKL, known biomarkers for necroptosis, in contrast to TNF-α/sMAC/zVAD (TSZ) treatment (Fig. 4h), indicating that CREB3-CF-induced karyoptosis is distinct from necroptosis. Additionally, CREB3-FL or CREB3-CF overexpression did not alter Gasdermin D protein levels, in contrast to the LPS-treated positive control group (Fig. 4i), suggesting that CREB3-CF-induced nuclear rupture is distinct from pyroptosis. Collectively, these findings demonstrate that CREB3-CF-mediated karyoptosis is a distinct cellular process that differs from apoptosis, canonical autophagy, necroptosis, and pyroptosis.

### ER stress triggers the cleavage of CREB3-FL to produce CREB3-CF via S1P and S2P at the nuclear membrane

**Fig. 5:**
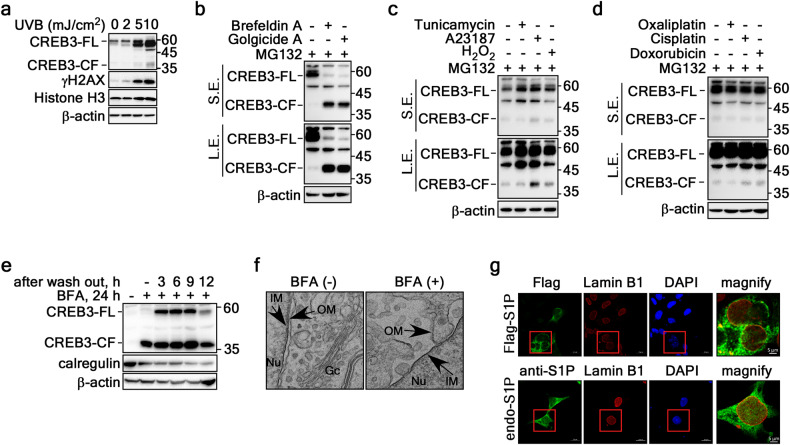
Identification of extrinsic stimuli that induce karyoptosis. **a** Western blot showing that UVB exposure increases the endogenous protein levels of CREB3-FL and CREB3-CF. **b** Western blot analyses revealing that ER stress, induced by vesicle trafficking inhibitors such as brefeldin A and golgicide A, leads to increased cleavage of CREB3-FL and subsequent production of CREB3-CF. **c** Western blot results indicating that ER stress, triggered by elevated levels of Ca^2+^ and ROS, moderately increases the protein levels of CREB3-FL and CREB3-CF, while the inhibition of glycosylation does not have this effect. **d** Western blot analyses showing that direct DNA damage caused by cisplatin and doxorubicin, but not oxaliplatin, slightly increases CREB3-CF protein levels without affecting CREB3-FL protein levels. **e** Western blot analyses illustrating the changes in the protein levels of CREB3-FL and CREB3-CF over time following washout of BFA. **f** TEM images demonstrating that BFA damages the nuclear membrane. **g** ICF image revealing that both overexpressed and endogenous S1P are localized not only to the cytoplasm but also to the nuclear membrane.

Although CREB3-FL, a type II membrane protein, is synthesized primarily at the ER and processed by site-1 protease (S1P) and site-2 protease (S2P) at the Golgi complex, it appears to be localized to and cleaved at the nuclear membrane (Fig. 1). Therefore, identifying external stimuli that induce CREB3-FL cleavage and CREB3-CF production is crucial. Notably, the levels of both CREB3-FL and CREB3-CF levels increased in response to UVB treatment in a dose-dependent manner (Fig. 5a). Additionally, ER stress-inducing vesicle trafficking inhibitors, such as brefeldin A and golgicide A, significantly enhanced CREB3-CF production while markedly decreasing CREB3-FL levels (Fig. 5b). Among the known ER stressors tunicamycin, A23187, and H_2_O_2_, the stressors A23187 and H_2_O_2_ (but not tunicamycin) elevated CREB3-CF levels (Fig. 5c). Intriguingly, all three stressors increased CREB3-FL protein levels (Fig. 5c). Direct DNA damage-inducing anticancer drugs such as oxaliplatin, cisplatin, and doxorubicin did not significantly alter CREB3-FL or CREB3-CF protein levels (Fig. 5d), suggesting that abnormal vesicular trafficking mediated by ER stress might contribute to CREB3-FL cleavage and subsequent CREB3-CF production, leading to karyoptosis. A brefeldin A treatment/washout experiment demonstrated that increased CREB3-CF levels persisted up to 9 h post-washout, whereas the decreased CREB3-FL by BFA was increased at 3 h, sustained until 9 h, and reduced at 12 h post-washout (Fig. 5e). This process correlated with BFA-induced ER stress and disassembly, as evidenced by the reduced calregulin levels (Fig. 5e) and by transmission electron microscopy observations (Fig. 5f). To identify potential proteases responsible for CREB3-FL cleavage into CREB3-CF, we conducted an extensive literature review and employed online prediction tools such as PeptideCutter and DNASTAR. These analyses indicated that the only potential proteases for the cleavage of CREB3-FL are S1P and S2P. Notably, no specific cleavage sites typically associated with proteases such as caspases, Factor Xa, granzyme B, hydroxylamine, protein-endopeptidase, thrombin, or tobacco etch virus protease were identified in CREB3. ICF analysis to determine the localization of overexpressed and endogenous S1P revealed that while S1P protease was detected mainly in the cytosol, it was also present in the nucleosol and nuclear membrane (Fig. 5g). These findings suggested that CREB3-FL cleavage and CREB3-CF production occur via S1P and S2P at the nuclear membrane with high efficiency.

### CREB3-CF-induced karyoptosis evokes DDR

**Fig. 6:**
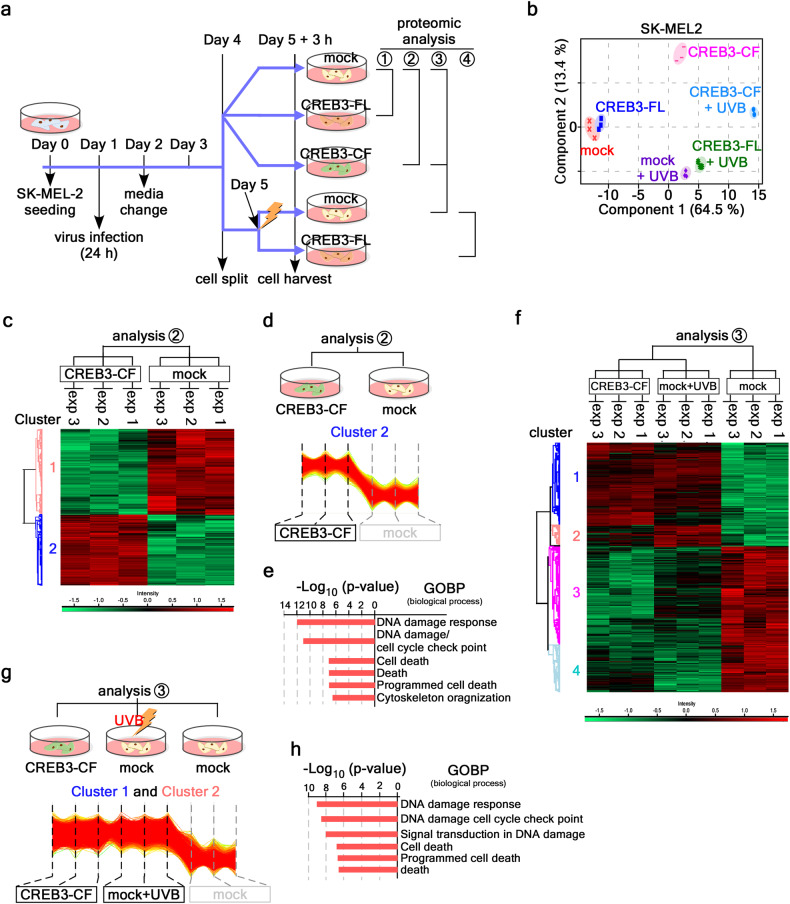
Proteomic analysis showing that CREB3-CF-induced karyoptosis also causes DDR-mediated cell death. **a** Illustration showing the sampling strategies used for proteomic analysis of CREB3-CF-induced karyoptosis. The comparisons used were as follows: analysis 1, mock and CREB3-FL; analysis 2, mock and CREB3-CF; analysis 3 CREB3-CF and mock+UVB, mock+UVB and mock, CREB3-CF and mock, and CREB3-CF/mock+UVB and mock; and analysis 4, mock+UVB and CREB3-FL + UVB. **b** Illustration of the distances of the protein sets in 5 different cells in Fig. 5a. **c** Hit map of CREB3-CF-induced karyoptosis according to analysis 2. Red, increase; green, decrease. **d** Protein frequency of the Cluster 2 proteins in CREB3-CF-induced karyoptotic cells according to analysis 2. **e** GO analysis of Cluster 2 proteins in CREB3-CF-induced karyoptotic cells according to analysis 2. A detailed list is provided in Supplementary Table 1. **f** Hit maps of CREB3-CF, mock+UVB, and mock to compare CREB3-CF and mock+UVB; mock+UVB and mock CREB3-CF and mock, and CREB3-CF/mock+UVB and mock according to analysis 3. Red, increase; green, decrease. **g** Illustration showing the protein frequency of Cluster 1 and 2 proteins in CREB3-CF-induced karyoptotic cells according to analysis 3. **h** GO analysis of Cluster 1 and 2 proteins in CREB3-CF-induced karyoptotic cells according to analysis 3. A detailed list is provided in Supplementary Table 2.

Since CREB3 interacts with Lamin A and B1, the interaction of CREB3 and Lamin at the nuclear inner membrane might play a key role in maintaining normal nuclear morphology by forming a complex with nuclear DNA and other nuclear envelope proteins^30^. Since the deformation of nuclear morphology is associated with human diseases via the DDR^31^, the maintenance of nuclear integrity is pivotal for maintaining cellular homeostasis^32^. Previous results demonstrated that UVB-induced abnormal nuclear morphology and cell death^33,34^ are associated with UV-induced DNA damage^35–37^ via Hutchinson–Gilford progeria syndrome (HGPS)-like abnormal nuclear shapes^38^ and cellular senescence^39,40^. Since CREB3-CF overexpression induced karyoptosis, which was associated with CREB3-FL anchoring and Lamin interaction at the nuclear inner membrane, a proteomic study induced by CREB3-CF might identify the factors and signaling cascades that induce the explosive nuclear membrane rupture. To identify the factors involved in karyoptosis and signaling cascades regulated by CREB3-CF, we designed an experimental strategy to compare protein profiles using SK-MEL-2 cells stably expressing mock, CREB3-FL, or CREB3-CF (Fig. 6a). The cells were split on day four and divided into two groups. After stabilization for 24 h (Day 5), one was continuously cultured without UVB stimulation, and the other was stimulated with 10 mJ/cm^2^ UVB (Fig. 6a). Lysates obtained on Day 5 and at 3 h after UVB treatment were subjected to proteomic analysis. A PCA plot indicating the distribution values of the differentially expressed proteins was generated to compare the responses of the different cells, and the results indicated that the mock and CREB3-FL-expressing cells were very similar (Fig. 6b). Moreover, when mock and CREB3-FL cells were irradiated with UVB, 65% of the proteins that were differentially expressed in both cell lines were similarly clustered with respect to component 1, as shown in Fig. 6b, indicating that CREB3-CF-induced karyoptotic proteins underwent similar changes in response to UVB irradiation in both mock and CREB3-FL cells (Fig. 6b). However, 13.4% of the component 2 clustered proteins were differentially expressed, indicating strong involvement in CREB3-CF-induced karyoptosis (Fig. 6b). Since CREB3-CF + UVB cells were shifted to the right from CREB3-CF cells on the *x*-axis, the changes in protein expression in CREB3-CF and CREB3-CF + UVB cells may play an additive role in karyoptosis and DDR-mediated cell death (Fig. 6b). A volcano plot of mock and CREB3-FL overexpression cells or mock+UVB and CREB3-FL + UVB cells did not reveal critical differences in the protein profile (Supplementary Fig. 3a, b). There were only nine induced and two reduced proteins (Supplementary Fig. 3a). In contrast, the mock and CREB3-CF comparisons showed dramatic differences in the protein profiles. Among the 4,519 proteins, the amounts of 2051 were increased in CREB3-CF-treated cells (Fig. 6c and Supplementary Table 1). The clustering data of these 2051 proteins from the CREB3-CF cohort were distinguishable from those from the mock cohort (Fig. 6d). Gene Ontology analysis of the cluster 2 proteins revealed that CREB3-CF significantly (according to *p* values) upregulated the expression of proteins involved in DNA repair and cell death compared to those in the mock control group (Fig. 6e and Supplementary Table 1). The ontological classification of these proteins was divided into six different pathways, DDR, DDR/cell cycle checkpoint, cell death, death, programmed cell death, and cytoskeletal organization. Next, we assessed whether UVB-induced DNA damage was similar to that induced by CREB3-CF. For this purpose, we prepared a lysate from UVB-irradiated mock cells (Fig. 6a). The results demonstrated that the overall pattern of increased protein expression in mock+UVB cells was similar to that in CREB3-CF-overexpressing cells (Fig. 6f). However, the CREB3-CF-expressing and mock+UVB-treated cells showed 560 differentially expressed proteins in six Gene Ontology categories, namely, the DDR, DNA damage cell cycle checkpoint, signal transduction in DNA damage, cell death, programmed cell death, and death (Fig. 6g, h and Supplementary Table 2). These results indicated that CREB3-CF initiated the karyoptosis signaling pathway in combination with the DDR, which was triggered by UVB, resulting in cell death.

### CREB3-CF-triggered karyoptosis blocks cell proliferation via cellular senescence

**Fig. 7:**
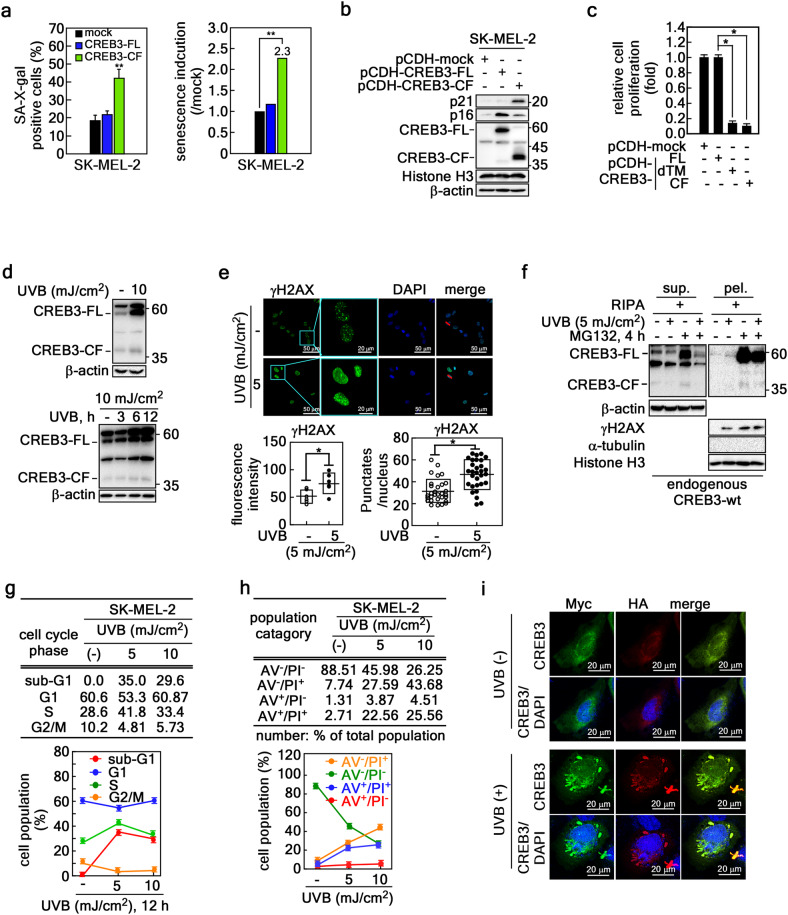
CREB3-CF-triggered karyoptosis blocks cell proliferation via cellular senescence. **a** Graphs showing cellular senescence induction by the expression of CREB3-FL or CREB3-CF. Left, percentage of β-gal-positive cells among more than 200 cells. Right, Fold increase in β-gal-positive cells among cancer cells. The cells stained with β-gal are shown in Supplementary Fig. 5a. **b** CREB3-CF induces p21 protein expression in SK-MEL-2 and HeLa cells. **c** Graphs showing that the expression of CREB3-CF or CREB3-dTM, but not CREB3-FL, suppresses the proliferation of SK-MEL-2 cells. The cells stained with crystal violet are shown in Supplementary Fig. 5b. **d** hat UVB increases CREB3-FL and CREB3-CF expression. **e** UVB induces DDR via the detection of nuclear puncta of γH2AX. **f** Illustration of the increase in nuclear DNA bound to endogenous CREB3-FL and -CF by UVB irradiation. **g** Cell cycle analysis data showed that the increase in UVB dose was not associated with sub-G1 enhancement. **h** Flow cytometry data showing that the early necrosis/necroptosis population is not associated with the late apoptosis/necrosis/necroptosis population. In contrast, UVB irradiation continuously decreased the live cell population in a dose-dependent manner. **i** Images of confocal microscopy images showing nuclear membrane ripping and rupture in CREB3-FL-overexpressing cells after UVB treatment.

Abnormal nuclear morphology caused by deformation of the nuclear membrane is associated with cellular senescence, as shown by HGPS^38,39^. CREB3-CF-induced karyoptosis similarly increased the senescence-associated β-gal-positive cell population and β-gal intensity (Fig. 7a and Supplementary Fig. 4a). The expression of biochemical markers involved in cellular senescence, including p21, was dramatically increased in CREB3-CF-overexpressing cancer cells (Fig. 7b). Notably, SK-MEL-2 cells stably expressing CREB3-CF or CREB3-dTM, but not CREB3-FL, exhibited severely reduced cell proliferation (Fig. 7c and Supplementary Fig. 4b). Based on the rationale that the proteomic study suggested a linkage between the signaling cascades of UVB-induced DDR (Figs. 2d, 4a, and 5) and CREB3-CF-induced karyoptosis, we further observed that UVB irradiation increased CREB3-FL and CREB3-CF (Fig. 7d), indicating that UVB might act as an extrinsic factor causing CREB3-CF cleavage and karyoptosis. The induction of DDR by UVB irradiation was confirmed by the increase in γH2AX intensity and puncta (Fig. 7e). Notably, UVB increased the levels of DNA-bound CREB3-FL and CREB3-CF in the pellet fraction but not in the soluble fraction (Fig. 7f). Surprisingly, MG132, a proteasomal degradation inhibitor, dramatically enhanced the levels of CREB3-FL and CREB3-CF protein in both the soluble and pellet fractions (Fig. 7f), indicating that CREB3-FL and CREB3-CF are extensively regulated by protein stability regulation and have a short half-life (Supplementary Fig. 4c). Cell cycle analysis revealed that a low dose of UVB (5 mJ/cm^2^) increased the proportion of cells in S phase and decreased the proportion of cells in G_1_ and G_2_/M phases (Fig. 7g). Interestingly, an increase in the dose of UVB (10 mJ/cm^2^) decreased the proportion of cells in S phase and restored the proportion of G_1_ phase cells to approximately what was observed in the UVB-untreated group (Fig. 7g). Moreover, we observed that the percentage of sub-G_1_ phase cells dramatically increased to 35% of the cell population in response to 5 mJ/cm^2^ UVB (Fig. 7g). Unexpectedly, 10 mJ/cm^2^ UVB had opposite effects on sub-G1 phase cells compared to 5 mJ/cm^2^ UVB (Fig. 7g), similar to previously reported findings^41^ indicating an unidentified type of cell death. We assessed the induction of cell death pathways by UVB using Annexin V (AV)/propidium iodide (PI) staining. We found that a low dose of UVB reduced the live cell population by approximately 42.5% (AV^−^/PI^−^). This decrease was associated with increases of approximately 20% in early necrosis/necroptosis (AV^−^/PI^+^), approximately 20% in late-phase cell death (also known as late apoptosis and necrosis/necroptosis, AV^+^/PI^+^), and approximately 2% in early apoptosis (AV^+^/PI^−^) (Fig. 7h), indicating that a low dose of UVB induced late-phase cell death via the early necrosis/necroptosis pathway. Importantly, when the UVB dose was increased to 10 mJ/cm^2^, the live cell population subsequently decreased by approximately 23.7% (45.98% *vs*. 26.35%), and the early necrosis/necroptosis population increased by approximately 16% (27.59% vs. 43.68%, Fig. 7h). Moreover, since the percentage of AV^+^/PI^+^ cells increased by approximately 3% (22.56% vs. 25.56%), almost all SK-MEL-2 cells were killed by 10 mJ/cm^2^ UVB (Supplementary Fig. 4d), and cell proliferation was stopped (Fig. 7c). We concluded that the cells arrested at the early necrosis/necroptosis phase continuously died and were discarded. Since the decrease of approximately 62.22% in the live cell population caused by 10 mJ/cm^2^ (88.51% vs. 26.25%) was not attributable to late-stage cell death (AV^+^/PI^+^), the maximum cell death ratio for karyoptosis via early necrosis/necroptosis reached approximately 43%. Importantly, confocal microscopy of SK-MEL-2 cells transiently expressing Myc-CREB3-FL-HA revealed that UVB irradiation evoked karyoptosis characteristics, including explosive nuclear membrane rupture, nuclear morphology changes, DNA herniation, and microsatellite nuclear formation, which correlated with nuclear membrane rupture and invagination of the nuclear membrane in the region of herniated DNA (Fig. 7i). These results demonstrated that UVB is an environmental factor that induces karyoptosis. Taken together, our results demonstrated that cell death in karyoptosis occurs by explosive nuclear rupture.

## Discussion

The regulation of nuclear integrity, including the assembly and disassembly of the nuclear membrane and nuclear shape maintenance, is a fundamental process for maintaining life. When cells are subjected to stress that causes DNA damage, such as ultraviolet or ionizing radiation, DNA damage response (DDR)-inducing anticancer drugs, or mechanical stress that induces nuclear fragility, the nuclear membrane ruptures and bulk nuclear DNA herniation occurs: these conditions are considered initial processes in karyoptosis, a recently proposed type of RCD associated with neurodegenerative disease progression^42^. Abnormal nuclear morphology resulting from dysregulated nuclear membrane dynamics is linked to laminopathies, which are disorders characterized by a wide array of clinically distinct phenotypes, including muscular dystrophy and progeria syndrome^43^. Additionally, these observations have important implications for cancer therapeutics. Strategies such as inducing karyoptosis and promoting cancer cell senescence could be viable approaches for cancer treatment (Fig. 7a and Supplementary Fig. 4a). Furthermore, the regulation of metastasis by controlling membrane flexibility during cell migration and the potential to influence cancer progression through the regulation of heterochromatin and euchromatin remodeling, thereby affecting gene expression, represent important areas of research with therapeutic relevance^43^. Although karyoptosis phenotypes include excessive excretion of irreplaceable nuclear components, resulting in abnormal nuclear morphology, loss of nuclear integrity, irreparable DNA damage, and atrophy of the cytoplasm^12,13^, the molecular initiators of karyoptosis have not yet been elucidated. Our results suggest a key event at the INM in which CREB3-FL may undergo enzymatic cleavage at the transmembrane region, likely via the S1P/S2P intramembrane protease (Fig. 5). PeptideCutter (https://web.expasy.org/peptide_cutter/) and DNASTAR analyses indicated that none of the specific cleavage sites typically associated with proteases such as caspase 1–10, factor Xa, granzyme B, hydroxylamine, protein-endopeptidase, thrombin, or tobacco etch virus protease were present in CREB3. Given the evidence that CREB1 is cleaved by caspase-6 and −8^44^ at the consensus amino acid sequence, cleavage of ILNDLSSD and CREB3-FL by caspases was also considered to be possible. However, CREB3 lacks this consensus sequence. Interestingly, BFA, an ER stressor that inhibits anterograde vesicle transport from the ER to the Golgi complex, increased the cleavage of CREB3-FL (Fig. 5b, e). TEM analysis clearly showed that BFA induced ONM damage (Fig. 5f). Moreover, our ICF results indicated that endogenous and overexpressed S1P were present not only in the cytoplasm but also in the nucleus and even in the nuclear membrane (Fig. 5g). These findings suggest that when cells are subjected to ER stress, CREB3-FL, which is located in the INM, may undergo cleavage by S1P and S2P. Further studies to identify the extrinsic stimuli that induce CREB3-FL cleavage and CREB3-CF production demonstrated that ER stressors, such as BFA, golgicide A, the calcium ionophore A23187, and UVB, induced CREB3-FL cleavage to produce CREB3-CF. However, tunicamycin and oxaliplatin did not induce CREB3-CF production. Additionally, H_2_O_2_, cisplatin, and doxorubicin induced weak CREB3-CF production (Fig. 5a–d). This cleavage untethered CREB3-FL from the nuclear membrane, resulting in the loss of equilibrated tension between the outward expansion force of packed DNA and the inward force of the nuclear membrane, triggering explosive nuclear membrane rupture, abnormal nuclear membrane folding, and rupture of the nuclear membrane at the side of the nucleus. In this process, exfoliated nuclear DNA herniated to the cytoplasm will eventually trigger cell death. We named this type of cell death as karyoptosis.

Our research conclusively demonstrated that two variants of CREB3, namely, CREB3-dTM and CREB3-CF, which cannot anchor to the INM, can induce karyoptotic nuclear fragility (Figs. 2, 3, and 4). These variants also impede cell proliferation (Fig. 7c and Supplementary Fig. 4b). Notably, neither CREB3-FL overexpression nor CREB3-CF overexpression altered the biomarkers associated with apoptosis, autophagy, necroptosis, or pyroptosis (Fig. 4). Consequently, identifying biomarkers associated with CREB3-CF-induced karyoptosis is crucial for assessing the efficacy of this process. Accordingly, we focused on analyzing the composition of extracellular vesicles (EVs) as potential biomarkers for karyoptosis. This hypothesis was based on the observation that cytoplasmic puncta in CREB3-CF-induced karyoptosis were co-stained with histones, DAPI, and CREB3 (Fig. 2d); LC3, DAPI, and CREB3 (Fig. 4f); and lamin B, DAPI, and CREB3 (Fig. 4c). Furthermore, TEM images revealed an increase in the presence of extracellular vesicles in SK-MEL-2 cells overexpressing CREB3-CF (Fig. 4g, right panels). Notably, CREB3-CF overexpression in HT-29 colon cancer cells induced the formation of budding EVs and multiple intracellular vesicles (Fig. 4g, left panels). Since the induction of karyoptosis by CREB3-CF is observable in various cell types, our findings strongly support the idea that EVs generated during karyoptosis could serve as potential biomarkers for this process. A combined analysis of future proteomic data from extracellular vesicles in combination with current proteomic data (Fig. 6) holds great promise for identifying biomarkers specific to karyoptosis.

Our comprehensive population analysis of nuclear fragility, as assessed via ICF assays, revealed that CREB3-FL and CREB3-CF exhibited karyoptosis-inducing potentials of approximately 27 and 82%, respectively. This discrepancy can be attributed to two plausible explanations: (1) CREB3-FL-induced nuclear fragility advances gradually and involves a two-step mechanism. Initially, CREB3-FL cleavage occurs, likely facilitated by S1P and S2P. Subsequently, nuclear envelope rupture occurs when a sufficient accumulation of CREB3-CF competes locally with endogenous CREB3-FL at the nuclear membrane. This cascade of events might ultimately be repaired. Therefore, a small proportion of cells exhibited abnormal nuclear morphology (Fig. 2f). (2) Conversely, overexpressed CREB3-CF directly competes strongly with endogenous CREB3-FL bound to chromatin at the INM. This leads to the widespread dissociation of CREB3-FL from the tethered nuclear membrane, resulting in the induction of widespread karyoptotic cell death (Fig. 2f). Karyoptosis involves a nuclear membrane integrity and is thus distinct from apoptosis, autophagy, necroptosis, and pyroptosis (Fig. 4). Since karyoptosis is currently defined by morphological characteristics, the identification of molecular biomarkers and elucidation of the molecular mechanism of karyoptosis are necessary in the future.

Our previous report indicated that UVB radiation triggers ER stress and elevates Ca^2+^ levels^45^. Additionally, certain chemotherapeutic agents, such as cisplatin and doxorubicin, in combination with ROS-inducing stimuli, such as H_2_O_2_, initiate not only apoptosis but also other forms of cell death, including autophagy and necroptosis^8^. Although numerous studies have linked UVB exposure to an increase in DNA damage-induced apoptosis, our findings revealed that UVB-induced cell death occurs via a unique process, namely, karyoptosis. We utilized UVB as a karyoptosis-inducing stimulus because (1) UVB radiation triggers distinct cellular responses based on dosage, (2) various UVB radiation doses can induce carcinogenesis or increase cell death, (3) UVB radiation induces ER stress and subsequent unfolded protein responses, and (4) UVB radiation elevates the cytosolic Ca^2+^ concentration and ROS production. Notably, we observed that a high dose of UVB (10 mJ/cm^2^) resulted in the unexpected death of approximately 16–43% of the cell population, which was not attributable to traditional apoptosis. At the molecular level, we discovered that maintaining a balanced tension at the nuclear inner membrane between the expansion force of tightly packed DNA and the constraining force of the nuclear membrane is critical for nuclear integrity. The explosive rupture of the nuclear membrane following CREB3-CF expression suggested that CREB3-CF overexpression increases γH2AX levels, linking karyoptosis to the DDR. Notably, CREB3-CF overexpression increased cellular senescence (Fig. 7a) and reduced foci formation (Fig. 7c), indicating that CREB3-CF suppresses cell proliferation. These effects were closely related to the observed increase in p21 (Fig. 7b) and decrease in p53 (Fig. 4a), suggesting that karyoptosis may utilize a p53-independent, p21-dependent signaling pathway. Further analysis of cell populations in terms of the cell cycle, sub-G_1_ phase, and cell death pathways revealed that the early initiation stage of karyoptosis might involve signaling pathways related to other types of regulated cell death, including apoptosis, necroptosis, and autophagy. Although the interactions among these regulated cell death mechanisms are not fully understood, UVB irradiation of CREB3-FL-overexpressing cells was able to induce karyoptosis (Fig. 7i), indicating the potential ability of this process to be manipulated. Thus, elucidating the molecular mechanisms of karyoptosis could have the potential to significantly improve the treatment of human cancer. Although the present study revealed only UVB and ER suppressors as extrinsic triggering stimuli, the findings imply that the regulatory mechanism of proteases that produce CREB3-CF through CREB3-FL cleavage could be harnessed to induce karyoptosis for cancer treatment.

### Supplementary information

Supplementary infotmations

Supplementary Table S1 and S2

Supplementaty Live Image S1

Supplementaty Live Image S2

Supplementaty Live Image S3
