# Supplementary material for: Dysregulated CREB3 cleavage at the nuclear membrane induces karyoptosis-mediated cell death
Source: Exp Mol Med. 2024 Mar 13;56(3):686–99. doi: 10.1038/s12276-024-01195-1 (PMC10985101; doi:10.1038/s12276-024-01195-1)

[Supplementary Information]

Dysregulated CREB3 cleavage at the nuclear membrane induces karyoptosis-mediated cell death

Ga-Eun Lee[1,2], Geul Bang[3], Jiin Byun[1,2], Cheol-Jung Lee[1,4], Weidong Chen[1,2], Dohyun Jeung[1,2], Hyun-Jung An[1], Han Chang Kang[1,2], Joo Young Lee[1,2], Hye Suk Lee[1,2], Young-Soo Hong[5], Dae Joon Kim[6], Megan Keniry[7], Jin Young Kim[3], Jin-Sung Choi[1], Manolis Fanto[8], Sung-Jun Cho[9], Kwang-Dong Kim[10], and Yong-Yeon Cho[1,2]

Contents:

1. Supplementary Figures: Supplementary Fig. 1 to 4

2. Supplementary Tables: Supplementary Table 1 and 2

3. Supplementary live Images: Supplementary Live Image 1 to 3

4. **Whole blots for Western blotting**

1. Supplementary Figures: Supplementary Fig. 1 to 4

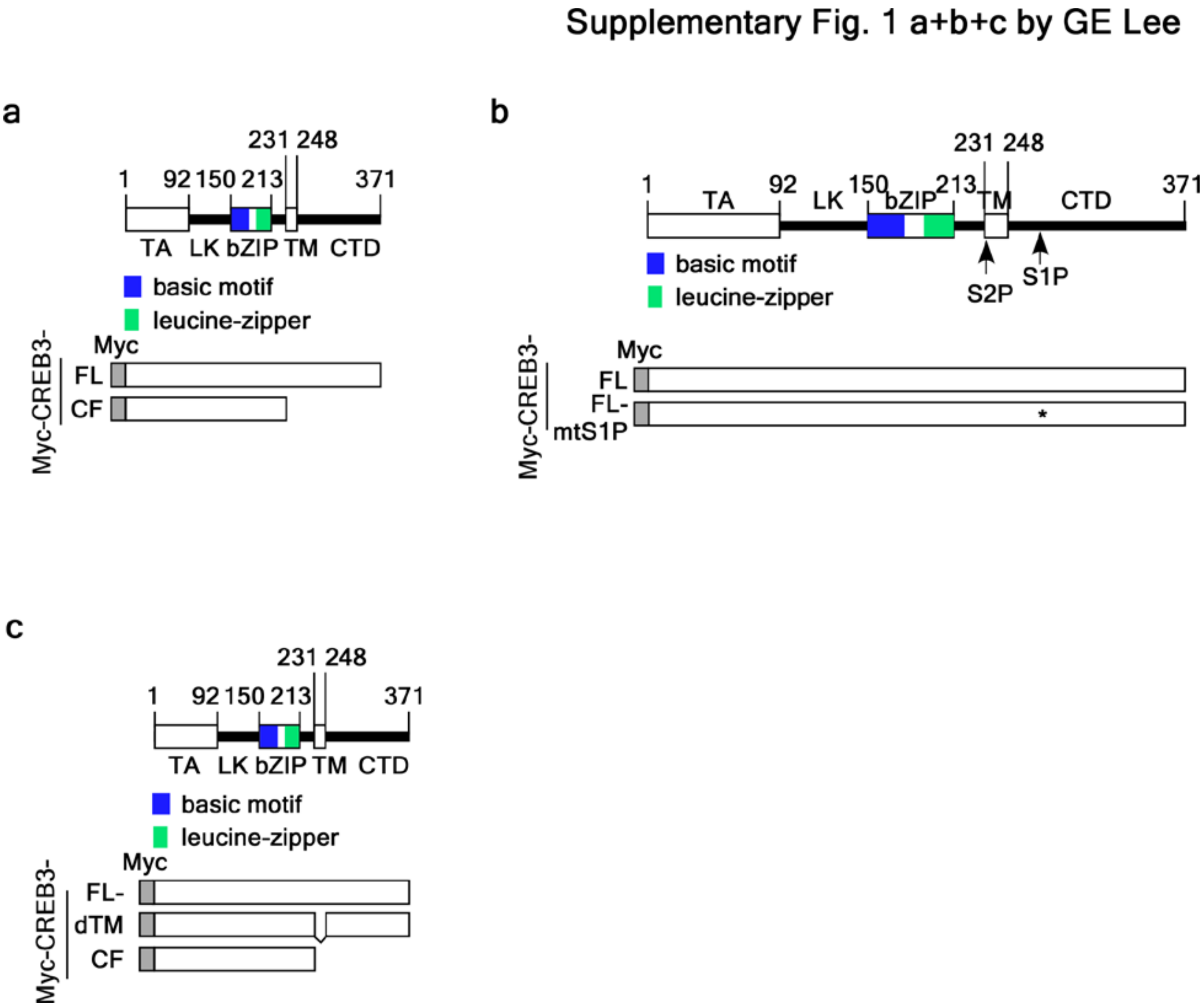

Supplementary Fig. 1 Vector maps for CREB3 using Fig. 1. a Vector map for CREB3-FL and CREB3-CF for overexpression. **b** Vector map for CREB3-FL and -FL-mtS1P. **c** Vector map for CREB3-FL, -dTM and -CF.

Supplementary Fig. 2 a+b by GE Lee

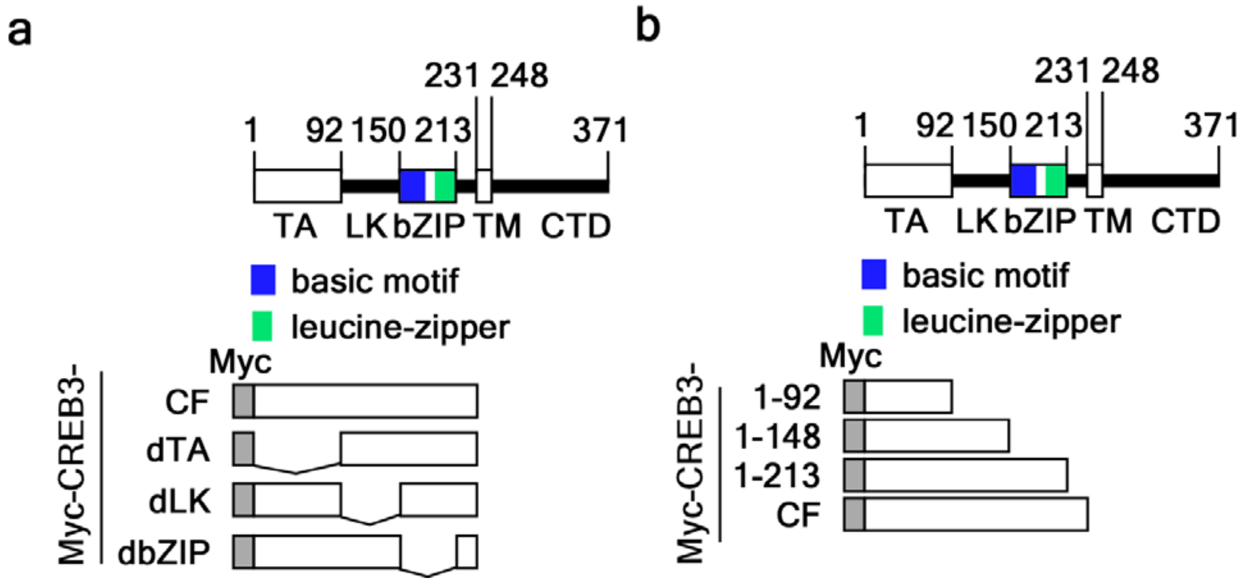

Supplementary Fig. 2 Vector maps for Fig. 2. The vector was used in Fig. 2 a, b, c, and f. **a** Vector maps for the domain deletion mutant of CREB3-CF. **b** Vector maps for serial deletion mutants of CREB3-CF for **Fig. 2c**.

Supplementary Fig. 3 a+b by GE Lee

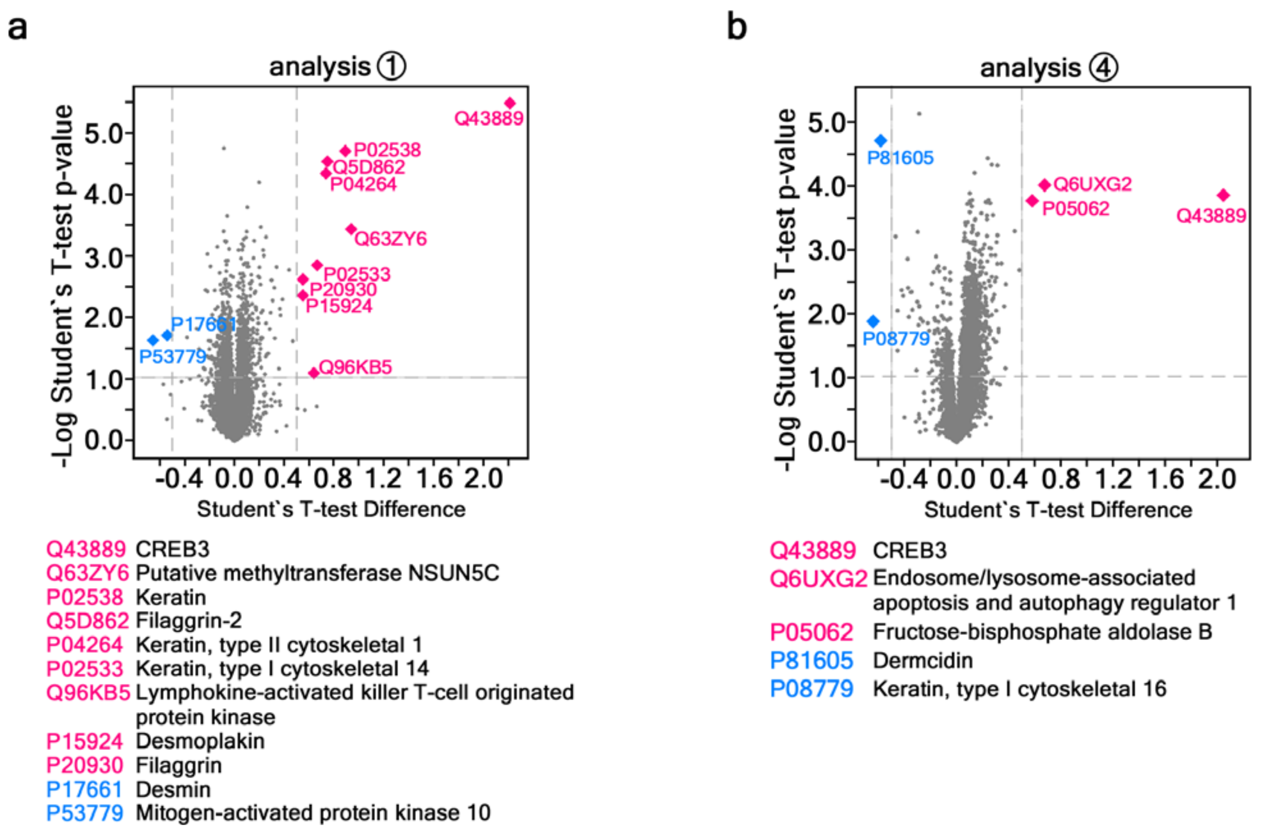

Supplementary Fig. 3 Proteomic analysis of CREB3-CF-induced karyoptosis evokes DDR-mediated cell death. a Illustration showing volcano plot of CREB3-CF-induced karyoptosis by analysis 1 illustrated in main **Fig. 6c**. Red, increase; blue, decrease. Detailed protein lists for 11 proteins are described. **b** Illustration showing volcano plot of CREB3-CF-induced karyoptosis by analysis 4 illustrated in main **Fig. 6f**. Red, increase; blue, decrease. Detailed protein lists for 5 proteins are described.

Supplementary Fig. 4 a+b+c+d by GE Lee

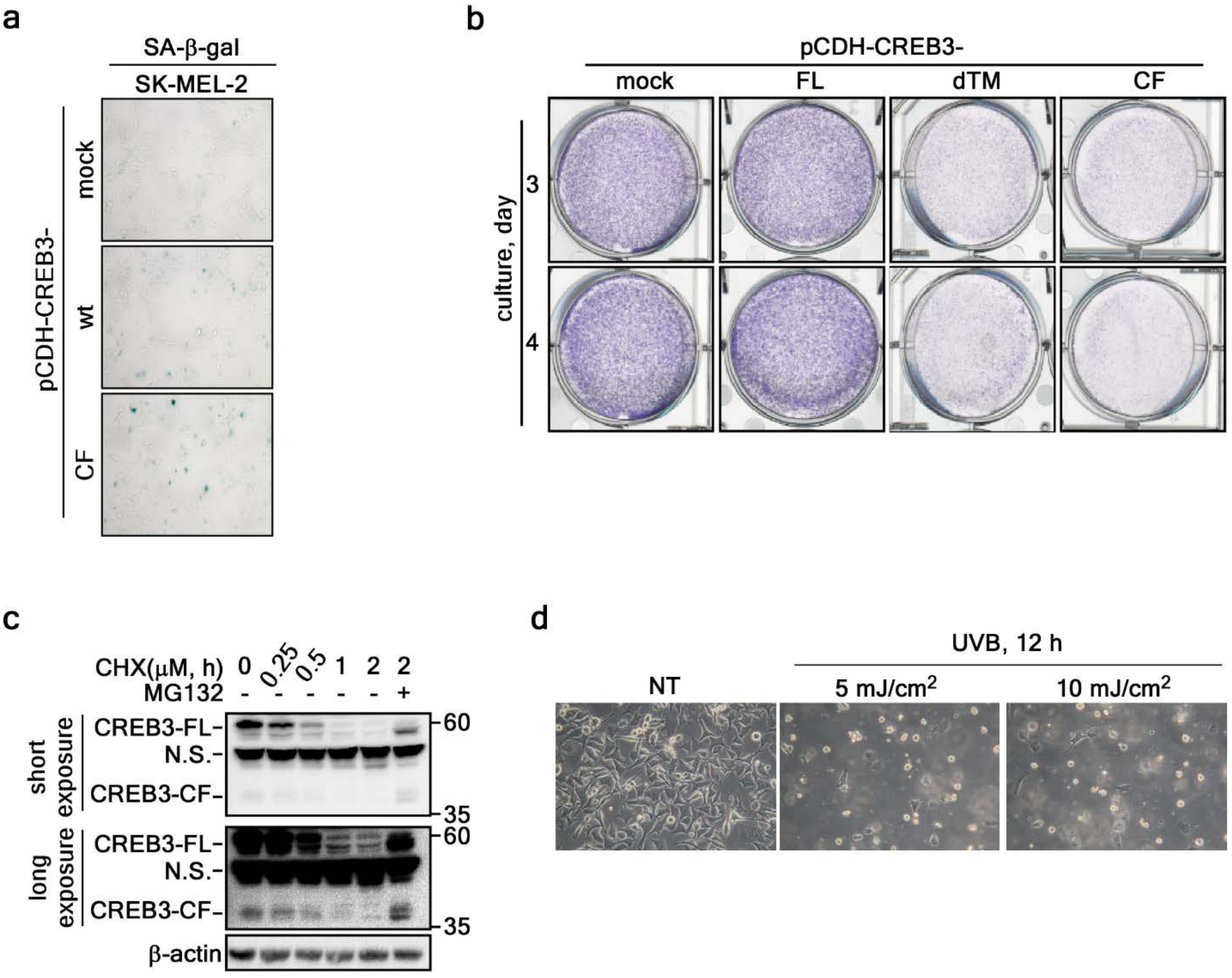

Supplementary Fig. 4 CREB-CF inhibits cancer cell proliferation by induction of cellular senescence. **a** Illustration showing that CREB3-CF expression increases cellular senescence by SA-β-gal staining in SK-MEL-2 and HeLa cells. **b** Illustration showing overexpression of CREB3-CF or -dTM suppresses cell proliferation in SK-MEL-2 cells, while cells stably expressing mock or CREB3-FL showed similar intensity of crystal violet staining. **c** Illustration showing half-life of CREB3-FL and -CF. **d** Illustration showing UVB-induced cell floating. Pictures were taken at 12 h after UVB irradiation with indicated doses.

2. Supplementary Tables: Supplementary Table 1 and 2

Supplementary Table 1. GOBP protein list obtained from proteomic analysis for mock and CREB3-CF (file name: Fig. 6e_proteomics-GOBP list).

Supplementary Table 2. GOBP protein list obtained from proteomic analysis for CREB3-CF, mock, and mock+UVB (file name: Fig. 6h_proteomics-GOBP list).

3. Supplementary live Images: Supplementary Live Image 1 to 3

Supplementary Live Image 1: Time-lapse Live Image obtained by Holotomography (HT-X1) of mock overexpression (file name: HT-X1-mock expression)

Supplementary Live Image 2: Time-lapse Live Image obtained by Holotomography (HT-X1) of CREB3-FL overexpression (file name: HT-X1-CREB3-FL expression)

Supplementary Live Image 3: Time-lapse Live Image obtained by Holotomography (HT-X1) of CREB3-CF overexpression (file name: HT-X1-CREB3-CF expression)

4. Supplementary Whole blots

Whole blot Fig. 1

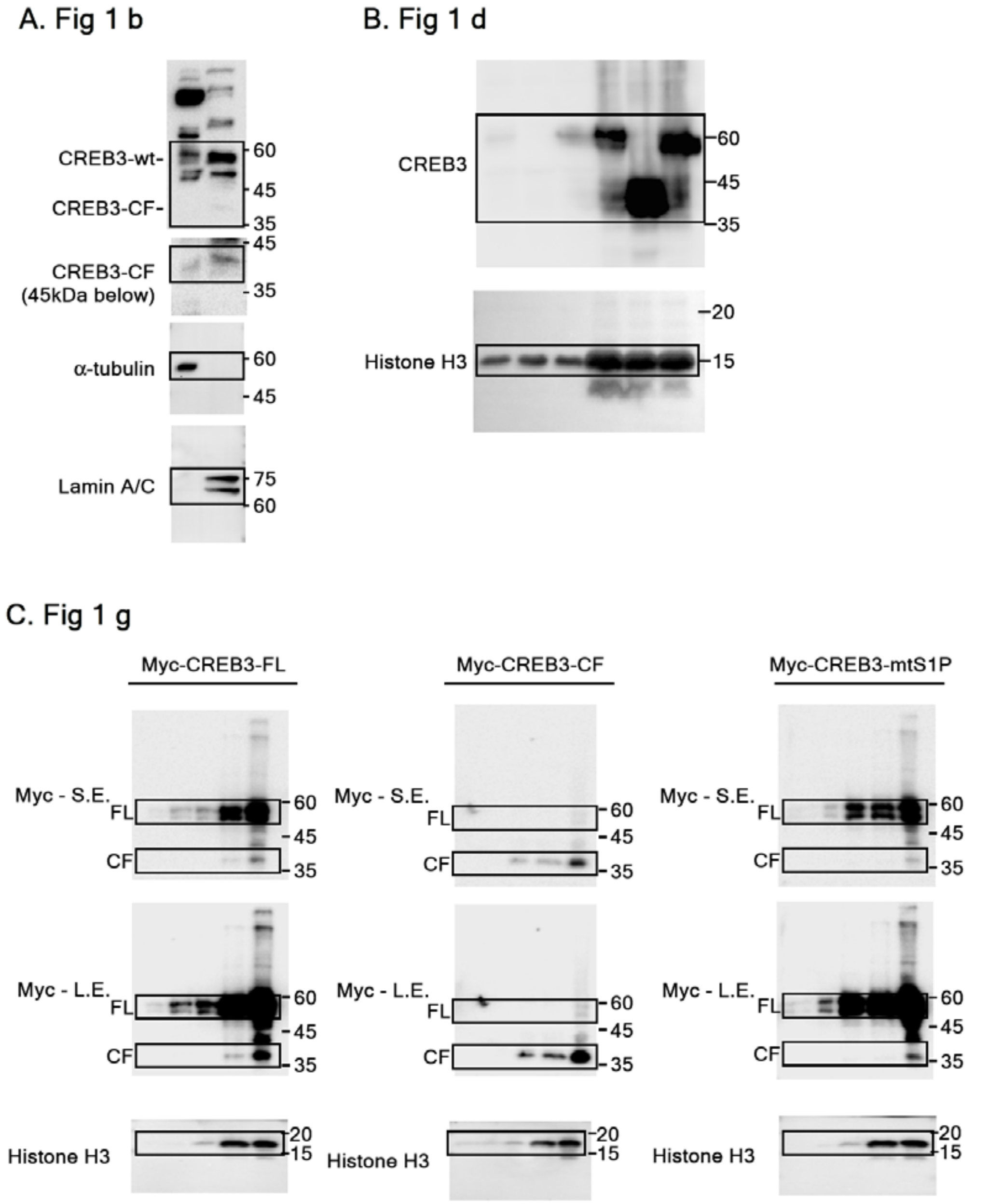

Whole blot Fig. 2

A. Fig 3 b

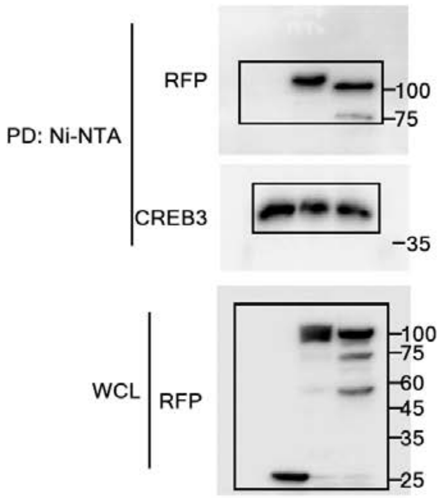

B. Fig 3 c

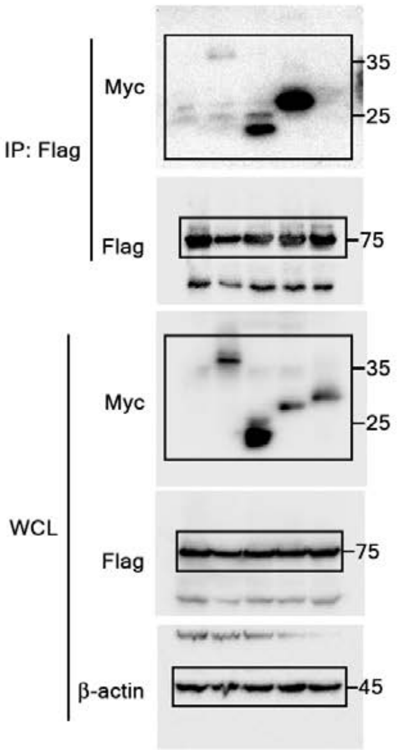

Whole blot Fig. 3

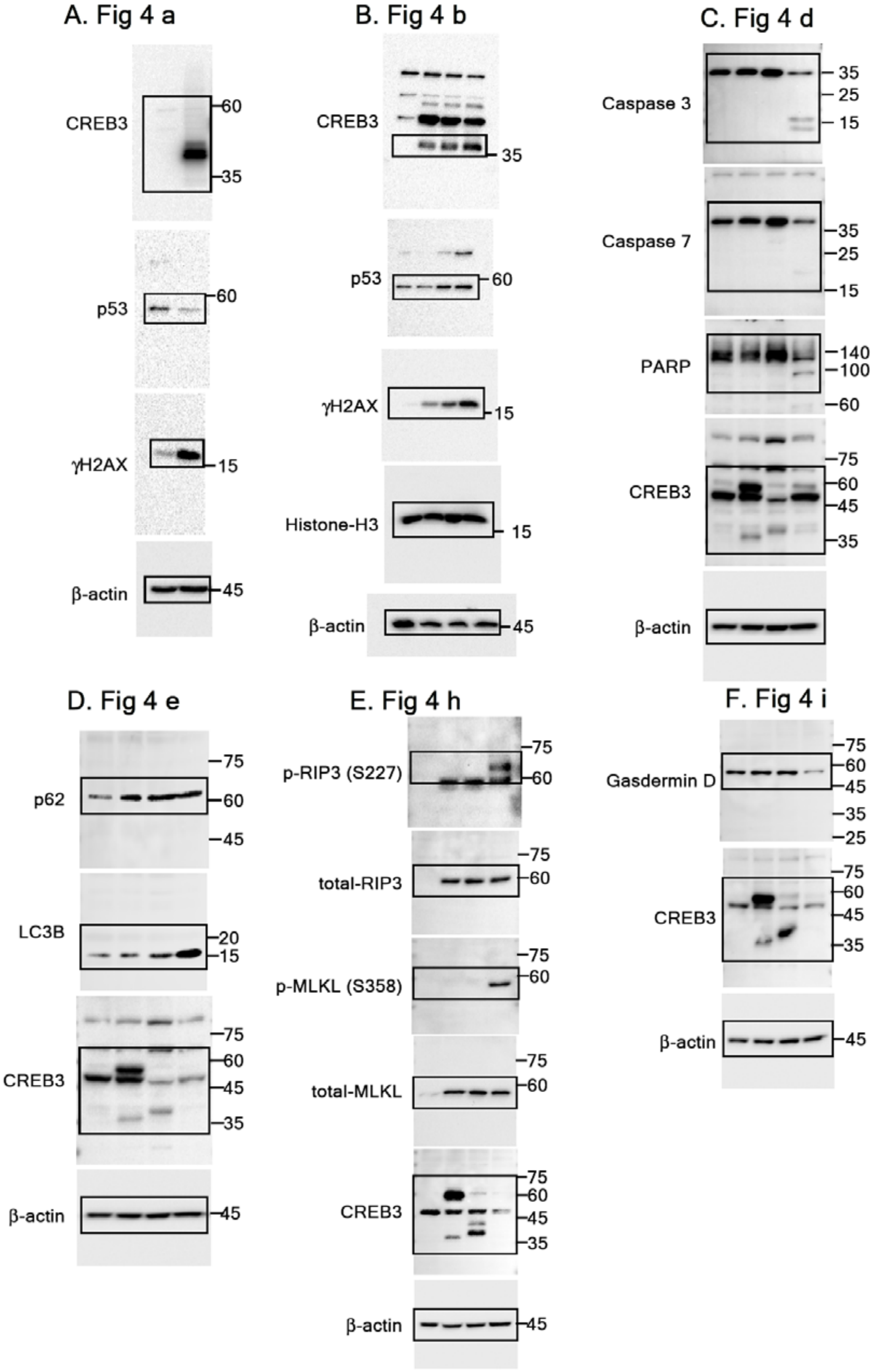

Whole blot Fig. 4

A. Fig 5 a

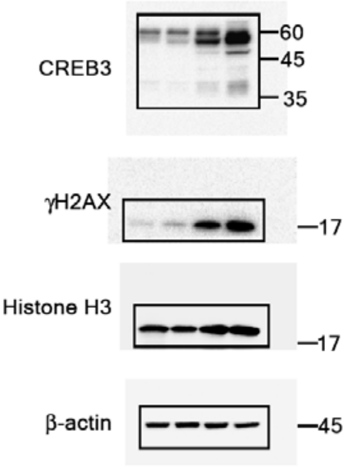

B. Fig 5 b

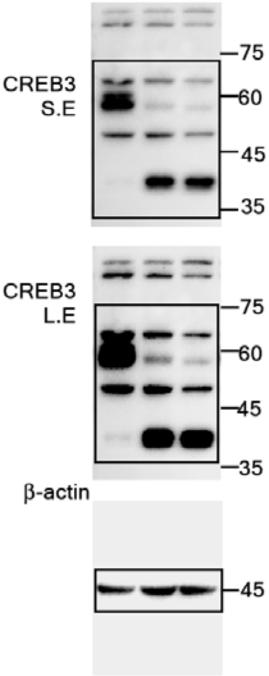

C. Fig 5 c

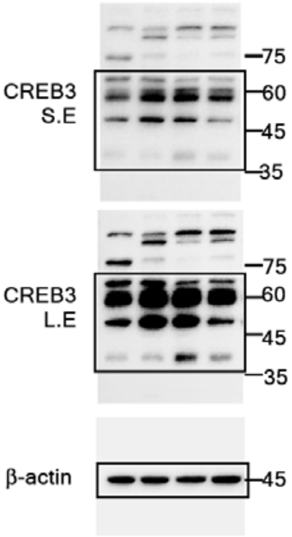

D. Fig 5 d

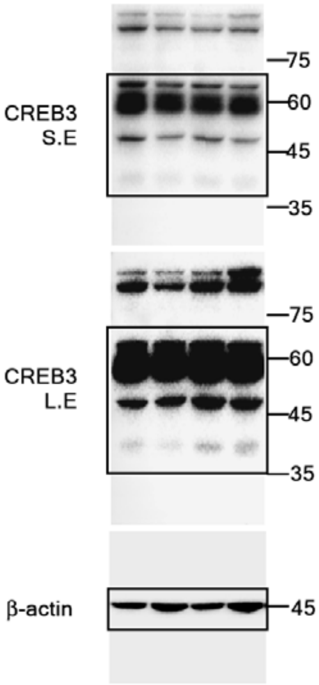

E. Fig 5 e

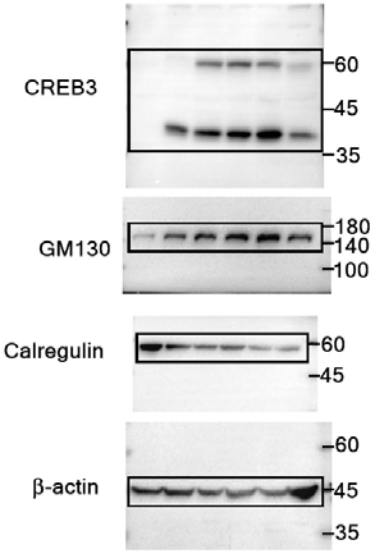

Whole blot Fig. 4

A. Fig. 7 b

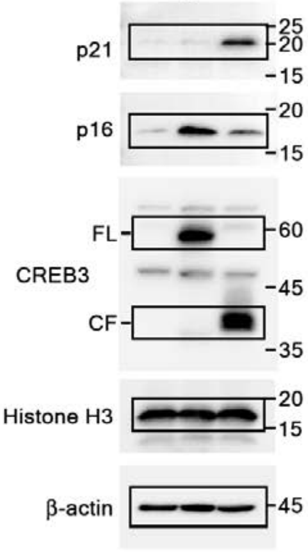

B. Fig. 7 d

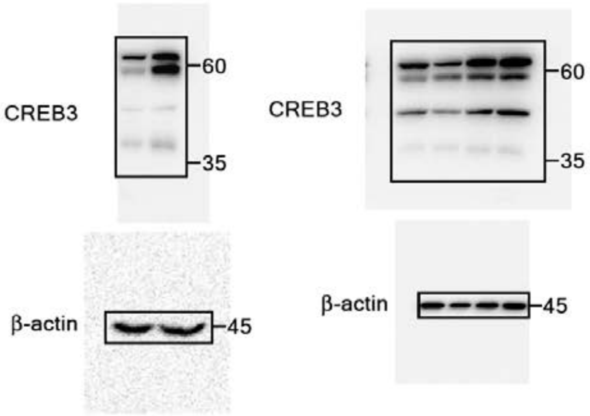

C. Fig. 7 f

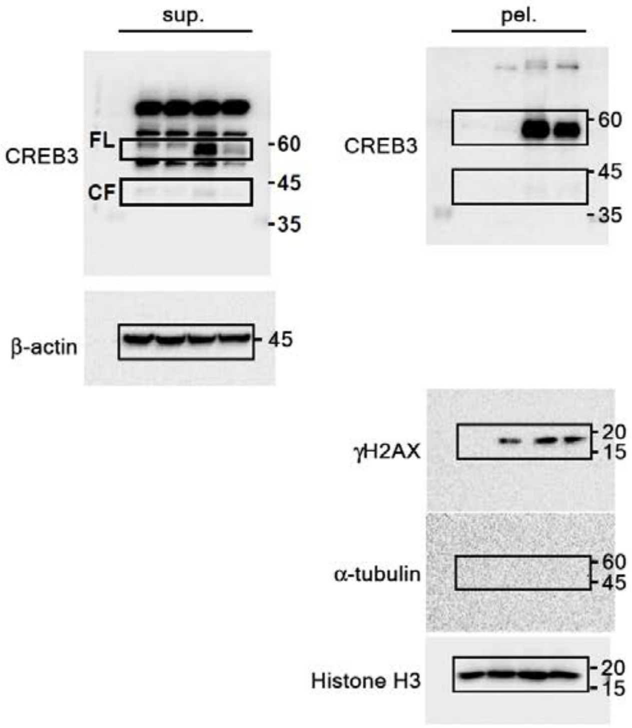

Supplementary Whole blot Fig. 1

A. Fig. S5 c

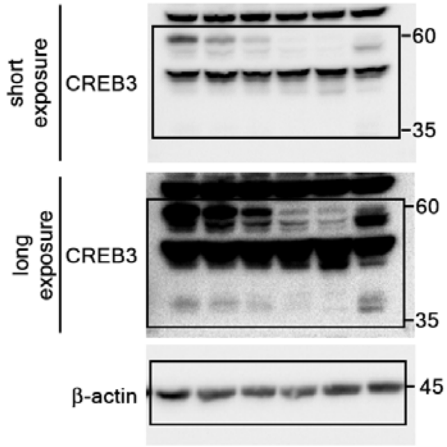

Supplement: Supplementary file 1 — Supplementary infotmations [file 12276_2024_1195_MOESM1_ESM.pdf]
